# Novel heterozygous truncating variant in *TUBB* associated with thrombocytopaenia and neurological abnormalities

**DOI:** 10.1186/s13023-026-04546-5

**Published:** 2026-08-13

**Authors:** Ilaria Svezia, Riccardo Zocchi, Michela Piccione, Giulia Ceglie, Giuseppe Palumbo, Matteo Becci, De Vito Rita, Jacopo Sartorelli, Teresa Rizza, Daniela Longo, Camilla Rossi Espagnet, Stefania Petrini, Emanuele Agolini, Antonio Novelli, Enrico Bertini, Francesco Nicita, Antonella Sferra

**Affiliations:** 1 Unit of Neuromuscular Disorders, Translational Pediatrics and Clinical Genetics, Bambino Gesù Children’s Hospital, IRCCS, 00165 Rome, Italy; 2 Department of Experimental Medicine, University of Rome Tor Vergata, 00133 Rome, Italy; 3 Research Laboratories, Bambino Gesù Children’s Hospital, IRCSS, 00165 Rome, Italy; 4 Haematology/Oncology, Cell and Gene Therapy, IRCCS Bambino Gesù Children’s Hospital, 00165 Rome, Italy; 5 Pathology Unit, Bambino Gesù Children’s Hospital, IRCCS, 00165 Rome, Italy; 6 Laboratory of Medical Genetics, Translational Cytogenomics Research Unit, Bambino Gesù Children’s Hospital, IRCCS, 00165 Rome, Italy; 7 Diagnostic and Interventional Neuroradiology Unit, Bambino Gesù Children’s Hospital, IRCCS, 00165 Rome, Italy

**Keywords:** *TUBB*, Microtubules, Thrombocytopaenia, Tubulinopathies, Proteasome, Kinesin

## Abstract

**Background:**

Tubulinopathies constitute a growing and heterogeneous class of disorders caused by pathogenic variants in genes encoding for tubulins, the structural components of microtubules. These conditions encompass a broad spectrum of neurological manifestations, most commonly including structural brain malformations and, less frequently, neurodegenerative disorders. In addition to brain diseases, tubulinopathies also include non-neurological conditions, such as female meiotic infertility and bleeding disorders. Among tubulin genes, *TUBB*, encoding the class I β-tubulin, has been associated with a wide range of clinical conditions, primarily including neurodevelopmental delay, structural brain abnormalities, and skin creases. More recently, *TUBB* has also been linked to inherited macrothrombocytopaenia, suggesting a broader, yet still incompletely defined, role of this β-tubulin in haematopoiesis and platelet biogenesis. In this study, we aim to investigate pathogenic mechanisms of a novel frameshift variant in *TUBB* (c.628dup; p.Ile210AsnfsTer40), identified in a subject affected by thrombocytopaenia and neurological involvement.

**Results:**

Functional analyses demonstrated that the p.Ile210AsnfsTer40 *TUBB* mutation leads to the production of a truncated β-tubulin with reduced protein stability and aberrant subcellular localization. The mutant protein showed impaired interaction with α-tubulin and kinesin family member 1 A (KIF1A), suggesting defective microtubule assembly and microtubule-mediated intracellular transport. Furthermore, the inhibition of the proteasome degradative pathway resulted in a marked increase of the mutant TUBB, indicating altered proteostasis and enhanced degradation of the truncated tubulin. Finally, we showed that the p.Asp249Val substitution in *TUBB*, previously associated with macrothrombocytopaenia, similarly reduces protein stability, weakens interaction with α-tubulin, and impairs TUBB incorporation into microtubules.

**Conclusions:**

This study associates the novel heterozygous truncating p.Ile210AsnfsTer40 variant in *TUBB* with a multisystem disorder characterized by haematological and neurological manifestations, expanding the phenotypic spectrum of *TUBB*-related diseases. Our findings indicate that reduced protein stability and functional impairment of β-tubulin represent key pathogenic mechanisms underlying this condition. Lastly, our experimental evidence further supports the implication of *TUBB* dysfunctions in haematological abnormalities, offering additional insight into the molecular basis and clinical variability of tubulin-related disorders.

**Supplementary Information:**

The online version contains supplementary material available at https://doi.org/10.1186/s13023-026-04546-5.

## Background

Microtubules are highly dynamic cytoskeletal structures continuously undergoing stochastic transitions between growth and shrinkage. They are composed of repeating subunits of different α- and β-tubulin isotypes, which are encoded, in humans, by a large multigene family [1].

Pathogenic variants in these genes have been linked to a wide range of diseases collectively known as tubulinopathies, which encompass a broad spectrum of neurological disorders most frequently including structural brain malformations [2–6] and rare neurodegenerative disorders [7–13]. In addition to brain diseases, tubulinopathies also include non-neurological conditions, such as female meiotic infertility and bleeding disorders [14–18].

Among bleeding disorders, mutations in *TUBB1*, *TUBB*,* TUBA8* and *TUBA4A* have been associated with congenital thrombocytopaenia in humans and mice. Intriguingly, most of these mutations acts through haploinsufficiency mechanisms, by reducing or causing the nearly complete absence of the mutant tubulin or impairing the formation of the tubulin heterodimer [14, 15, 19–27].

In this study, we report on the novel heterozygous truncating p.Ile210AsnfsTer40 mutation in *TUBB* identified in a patient affected by thrombocytopaenia and neurological involvement.

We demonstrated that this pathogenic variant leads to the formation of a less stable truncated tubulin, which undergoes proteasomal degradation. We also showed that the mutant tubulin is not incorporated into microtubules, presenting an aberrant nuclear localization, and is defective for α-tubulin and KIF1A interaction.

Therefore, our data partially support a loss of function mechanism of the disease-causing mutation that is consistent with haploinsufficiency, since it results in the apparent loss of tubulin functions and stability.

Our study also showed that the p.Asp249Val substitution in *TUBB*, recently associated with macrothrombocytopaenia [27], affects tubulin protein stability and affinity for α-tubulin, thus acting with pathogenic mechanisms that overlap with those of the p.Ile210AsnfsTer40 mutation.

In conclusion, the present study extends the spectrum of phenotypic abnormalities associated with *TUBB* mutations and supports a model in which reduced tubulin function and stability may contribute to thrombocytopaenia in humans. Moreover, our findings suggest that additional pathogenic mechanisms, such as proteostasis stress and nuclear accumulation of mutant tubulin, may also contribute to disease pathogenesis.

## Methods

### Study design and setting

This study describes a combined clinical and genetic analysis of a single patient affected by a complex multisystem disorder, together with a functional molecular investigation of the underlying mutation.

### Molecular analysis

After obtaining informed consent from the patient’s parents for genetic testing, peripheral blood samples were collected from the affected subject and the unaffected parents, and genomic DNA was extracted from circulating leukocytes. Enrichment and parallel sequencing were then performed utilizing a custom clinical exome panel, containing more than 8500 genes. Library preparation was carried out by using the Twist enrichment kit, according to the manufacture’s protocol (Twist Bioscience, South San Francisco, CA, USA), and sequenced on a NovaSeq 6000 (Illumina, Inc., San Diego, CA, USS) platform. The BaseSpace pipeline (Illumina, https://basespace.illumina.com/) and Geneyx software (Geneyx Genomex Ltd) were used for variant calling and annotating variants, respectively. Sequencing data were aligned to the hg19 human reference genome. Variants were analysed in silico by using Combined Annotation Dependent Depletion V.1.3, Sorting Intolerant From Tolerant, Polymorphism Phenotyping v2 and Mutation Taster for the prediction of deleterious non-synonymous SNVs for human diseases. Variants were examined for coverage and Qscore (minimum threshold of 30) and visualized by the Integrative Genome Viewer.

### Plasmid constructs

Wild-type (WT) MYC-tagged TUBB expression vector was purchased from Sino Biological Inc. (Beijing, China) (HG11626-NM). Nucleotide changes resulting in the p.Ile210AsnfsTer40 and p.Asp249Val mutations were introduced by site-directed mutagenesis (200523, Agilent Technologies, Santa Clara, CA). The entire transgene-coding regions were sequence validated following maxiprep yields (12162, Qiagen, Hilden, Germany).

### Cell culture and transfection

Human embryonic kidney 293 (HEK-293) and COS-1 cells were cultured as previously described [13]. HEK-293 and COS-1 cells were seeded in 6-well plates and transfected after 24 h, using Lipofectamine 2000 (11668-019, Thermo Fisher Scientific, Waltham, MA, USA) according to the manufacturer’s instructions.

### Protein stability assay

24 h after transfection, HEK-293 cells were treated with 100 µg/ml of cycloheximide (CHX) (C4859, Sigma-Aldrich, St. Louis, MO, USA) or with an equivalent volume of DMSO (untreated) (D5879, Sigma-Aldrich). Cells were harvested for protein extraction after 0, 8, 16, and 24 h of treatment or after 0 and 10 h. For combined MG132/CHX treatments, 24 h after transfection, HEK-293 cells were incubated with 20 µM MG132 (C2211, Sigma-Aldrich) for 8 h, followed by treatment with 100 µg/ml CHX for 15 h. Cells were harvested for protein extraction after MG132 and MG132/CHX treatment.

### MG132 treatment

24 h after transfection, HEK-293 cells were treated with 20 µM MG132 (C2211, Sigma-Aldrich) or with an equivalent volume of DMSO (untreated) for 8 h, as previously described [13].

### Protein extraction, co-immunoprecipitation experiment and immunoblot analysis

COS-1 cells were collected 24 h after transfection and HEK-293 cells were collected 24 h after transfection, 8 h after MG132 administration, or at different time points after CHX treatment and combined MG132/CHX treatment. Cells were lysed on ice with RIPA buffer (R0278-50ML, Sigma-Aldrich), as reported by [13]. For HEK-293 cells treated with MG132, the cell pellet obtained after centrifugation and corresponding to the detergent insoluble fraction, was resuspended with Laemmli buffer, using the same volume of the supernatant, corresponding instead to the detergent soluble fraction. 30 micrograms of protein were resuspended with Laemmli buffer. Protein samples were loaded and run on a 4–12% mini protein gel (NW04122BOX, Thermo Fisher Scientific) and transferred to a PVDF membrane (IPVH00010, Immobilon, Merck Millipore, Burlington, MA, USA).

For co-immunoprecipitation experiments, transfected HEK-293 cells were lysed as previously described [11]. One thousand five hundred micrograms of proteins were immunoprecipitated for 1 h with anti-c-Myc magnetic beads (88842, Thermo Fisher Scientific), previously incubated for 1 h with bovine serum albumin (BSA) (fraction V, 10775835001, Roche Diagnostics International AG, Rotkreuz, Switzerland) to minimize non-specific binding. Immunoprecipitated samples were subjected to immunoblot analysis, as described above.

### RNA extraction and quantitative real-time PCR

Total mRNA from transiently transfected HEK-293 cells and primary fibroblasts was isolated using Total RNA purification Kit (48300, Norgen Biotek Corp., Thorold, ON, Canada), according to the manufacturer’s protocol. One microgram of each RNA sample was reverse transcribed with the LunaScript RT Supermix kit (E3010L, New England Biolabs, Ipswich, MA, USA). Expression levels of *TUBB* mRNA were measured by quantitative real-time PCR (qRT-PCR) in an ABI PRISM 7500 Sequence Detection System (Life Technologies, Carlsbad, CA, USA) using Power SYBR^®^ Green I dye chemistry (2407657, Thermo Fisher Scientific).

For the qRT-PCR experiments in transfected HEK-293 cells we used a Forward primer annealing to the polylinker region of *TUBB* construct and a Reverse primer annealing in the exon 3 of *TUBB*, allowing the specific amplification of construct-derived transcripts. For qRT-PCR analyses in primary fibroblasts, we used a Forward primer located in the 5′ untranslated region (5′UTR) and a Reverse primer located across exon 1-exon 2 of *TUBB*.

Data were analysed using the ΔΔCt method with Phosphoglycerate Kinase 1 (PGK1) as housekeeping gene and data are shown as fold change relative to control. Notably, the use of an alternative housekeeping gene such as the hypoxanthine guanine phosphoribosyltransferase gene did not modify the data obtained with *PGK1* gene.

The primer sequences used for gene expression analysis in HEK-293 cells and primary fibroblasts are listed in Tables S1 and S2, respectively.

### Antibodies used for immunoblotting

For immunoblotting, following primary antibodies were used: anti-MYC (sc-40, Santa Cruz Biotechnology, Dallas, TX, USA; 1:500), anti-KIF1A (180153, Abcam, Cambridge, UK; 1:1000), anti-α-tubulin (#9099, Cell Signalling, Danvers, MA, USA; 1:2000), anti-β-actin (A2228, Sigma-Aldrich, 1:2000), anti-GFP (ab6556, Abcam, 1:1000). For the detection, 1:10.000 secondary antibodies goat anti-rabbit (111-035-003, Jackson Immuno Research, West Grove, PA, USA) and goat anti-mouse (115-035-146, Jackson Immuno Research) were used.

### Immunofluorescence analysis

COS-1 and HEK-293 cells were fixed with ice–cold methanol for 5 min, blocked with 5% BSA (A8022-100G, Sigma-Aldrich) for 1 h and incubated overnight with the primary mouse anti-MYC antibody (sc-40, Santa Cruz Biotechnology, 1:500). For MYC/α-tubulin double immunostaining, COS-1 cells were fixed with ice-cold methanol for 5 min, blocked with 3% BSA for 1 h and incubated overnight with a mixture containing the mouse anti-MYC antibody (sc-40, Santa Cruz Biotechnology, 1:500) and the rabbit anti-α-tubulin antibody (MA5-38163, Invitrogen, 1:100).

After primary incubation, cells were washed three times with PBS and incubated for 1 h with a secondary goat anti-mouse antibody conjugated to Alexa Fluor 555 (1:500, A21425, Invitrogen) or with a mixture containing a secondary goat anti-mouse antibody conjugated to Alexa Fluor 555 (1:500, A21425, Invitrogen) and a secondary goat anti-rabbit antibody conjugated to Alexa Fluor 488 (1:500, A11008, Invitrogen). Cells were then washed three times with PBS and incubated with Hoechst (H3570, Invitrogen; 1:10.000,) to counterstain nuclei. Confocal microscopy was performed on an Olympus FV3000laser-scanning confocal microscope (Evident Scientific, Hamburg, Germany) equipped with 405 nm and 561 nm laser sources.

### Data analysis

Statistical analysis was performed using GraphPad Prism 10.2.1 software (San Diego, CA, USA). Data are presented as mean ± standard error of the mean (SEM). Comparisons between two groups were performed using the Mann–Whitney U test, after confirming that data were not normally distributed by the Shapiro–Wilk test.

## Results

### Clinical assessment and molecular analysis

The patient is a 5-year-old boy, born at term after an uncomplicated pregnancy. The mother is a carrier of β-thalassemia. At birth, Apgar scores and auxological parameters were normal. Disease onset occurred at four months with severe anaemia, reticulocytopenia, and neutropenia. Initial blood examination, carried out in another hospital, showed haemoglobin 5.6 g/dL, white blood cells 4,420/mm³, neutrophils 590/mm³, and platelets 195,000/mm³. During hospitalization, ultrasound revealed two subcutaneous cysts in the left gluteal region, Bone marrow biopsy documented erythroid hyperplasia (granulocyte/erythroid ratio 1:1, cellularity 55–60%). Abdominal ultrasound was normal (spleen 5.5 cm). The patient received two transfusions with haemoglobin improvement, and vitamin B12 deficiency was treated with monthly intramuscular injections. At discharge, blood counts normalized.

At seven months of age, he manifested pallor and poor intake and was referred to our Institution. Full blood count showed he 7.4 haemoglobin/dL, mean corpuscular volume 83.4 fL, white blood cells 9,060/mm³, neutrophils 1,780/mm³, lymphocytes 6,750/mm³, platelets 173,000/mm³, and reticulocytes 35,000/mm³. Haemoglobin variant screening performed by HPLC showed normal HbA2 (2.6%) and slightly elevated HbF (10.5%) as it is expected for age. The normal HbA2 level argues against a clinically significant contribution of β-thalassemia to the patient’s anaemia. During hospitalization, haemoglobin declined progressively, requiring multiple red blood cells transfusions. Persistent decrease in platelets count was registered, reaching values around 80,000–90,000/mm³; transient moderate neutropenia occurred but resolved spontaneously. Bone marrow studies showed reduced erythroid and megakaryocytic populations with hypo-dysmegakaryocytopoiesis, karyotype normal. Extensive testing (microbiological, cardiological vitamin B12, eADA, DEB test) was unrevealing; erythropoietin was 500 mIU/mL. Neurological examination revealed non paralytic divergent strabismus of the right eye, binocular horizontal pendular nystagmus and mild axial hypotonia; an ophthalmologic evaluation showed diffuse retinal hypopigmentation. Brain magnetic resonance imaging (MRI) evidenced lower cerebellar vermis dysplasia.

The child was discharged, requiring repeated red blood cell transfusions until 14 months, then haemoglobin stabilized around 10.5 g/dL. Neutrophils normalized, but progressive asymptomatic thrombocytopaenia became evident (nadir 30,000/mm³) with normal platelet volume. Haematological parameters trend over time is reported in Table S3.

**Fig. 1:**
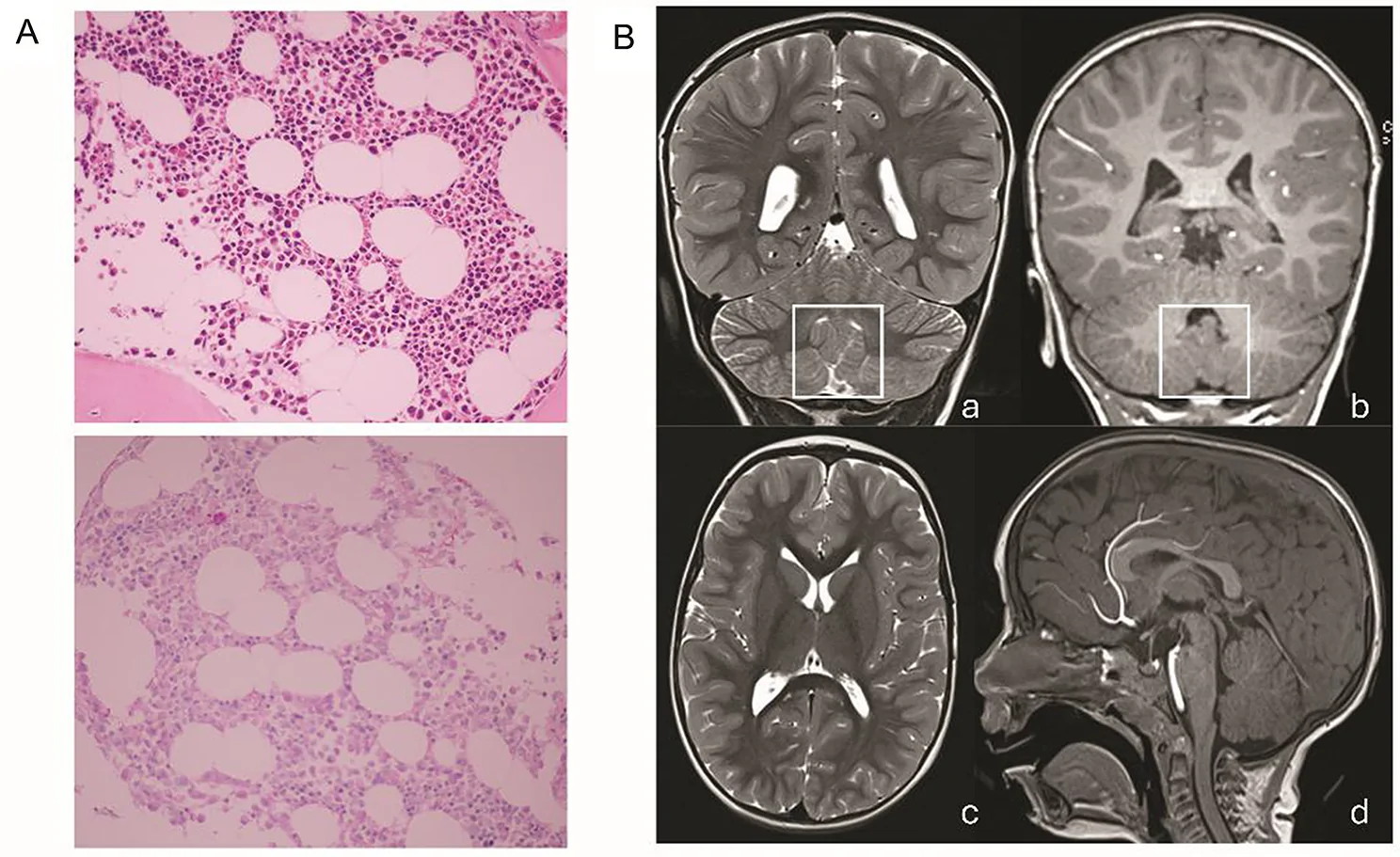
Haematological and neuroimaging features. (**A**) Haematoxylin and eosin-stained section of bone marrow biopsy shows a hypocellular marrow (upper panel). Immunohistochemistry of bone marrow section for CD61 highlights scarce megakaryocytic precursors (lower panel). (**B**) Brain MRI (A T2-weighted coronal view; B T1-weighted coronal view; C T2-weighted axial view; D T1-weighted sagittal view) of the patient at age 3 years showing dysplasia of the inferior cerebellar vermis (square in a and b), no abnormalities of basal ganglia and brainstem (c and d), thickness of the isthmus of the corpus callosum at the lower limits for age (d)

Immunologic workup was normal, but anti-GPIIb/IIIa, anti-GPIa/IIa, and anti-GPIb/IX antibodies resulted positive after initial negative results. Repeat bone marrow biopsy showed hypocellular dyshaematopoiesis with marked evident hypo-dysmegakaryocytopoiesis (Fig. 1A).

Additional investigations were performed on bone marrow aspirate, including conventional karyotyping, FISH analysis, and chromosomal microarray analysis, all of which yielded normal results.

Next generation sequencing for inherited anaemia and bone marrow failure (Table S4) was negative. Multiple therapeutic attempts (IVIG, steroids, Eltrombopag) were unsuccessful. At age 4, following influenza A, he developed petechiae with PLTs 9,000/mm³, requiring hospitalization and IVIG. At discharge, platelets remained low (15,000/mm³), though bleeding symptoms were absent.

During the following years, neurodevelopment milestones were acquired in normal ranges, but signs of atypical social interaction and behaviour appeared over time. Serial neurological examinations disclosed no additional signs of limb or gait ataxia, while nystagmus persisted but improved significantly. Serial ophthalmological examinations revealed no more abnormalities of the anterior and posterior eye segments. A new brain MRI at age 3 years confirmed the cerebellar vermis dysplasia and evidenced a mild thinning of the corpus callosum (Fig. 1B). A progressive lymphatic malformation in the right gluteal region was treated with sclerotherapy at age three years. Following treatment, an increase in its size was noted.

To identify the molecular event underlying the phenotype, exome sequencing was performed in the proband and unaffected parents, revealing the *de novo* heterozygous c.628dup (p.Ile210AsnfsTer40) variant in *TUBB* (NM_178014.4; UniProt ID P07437), not observed in population databases (gnomAD) (Figure S1). Exome analysis also identified the heterozygous *HBB* c.92 + 6T > C variant, inherited from the mother, consistent with β-thalassemia carrier status.

The patient’s clinical and haematological presentation was not suggestive of β-thalassemia. In particular, HbA2 levels were within the normal range (2.6% by HPLC) and MCV values remained normal throughout follow-up, arguing against a clinically significant contribution of β-thalassemia to the anaemia. No additional *HBB* variants were identified by exome sequencing, although this approach may not detect all genetic alterations affecting this gene. 

**Fig. 2:**
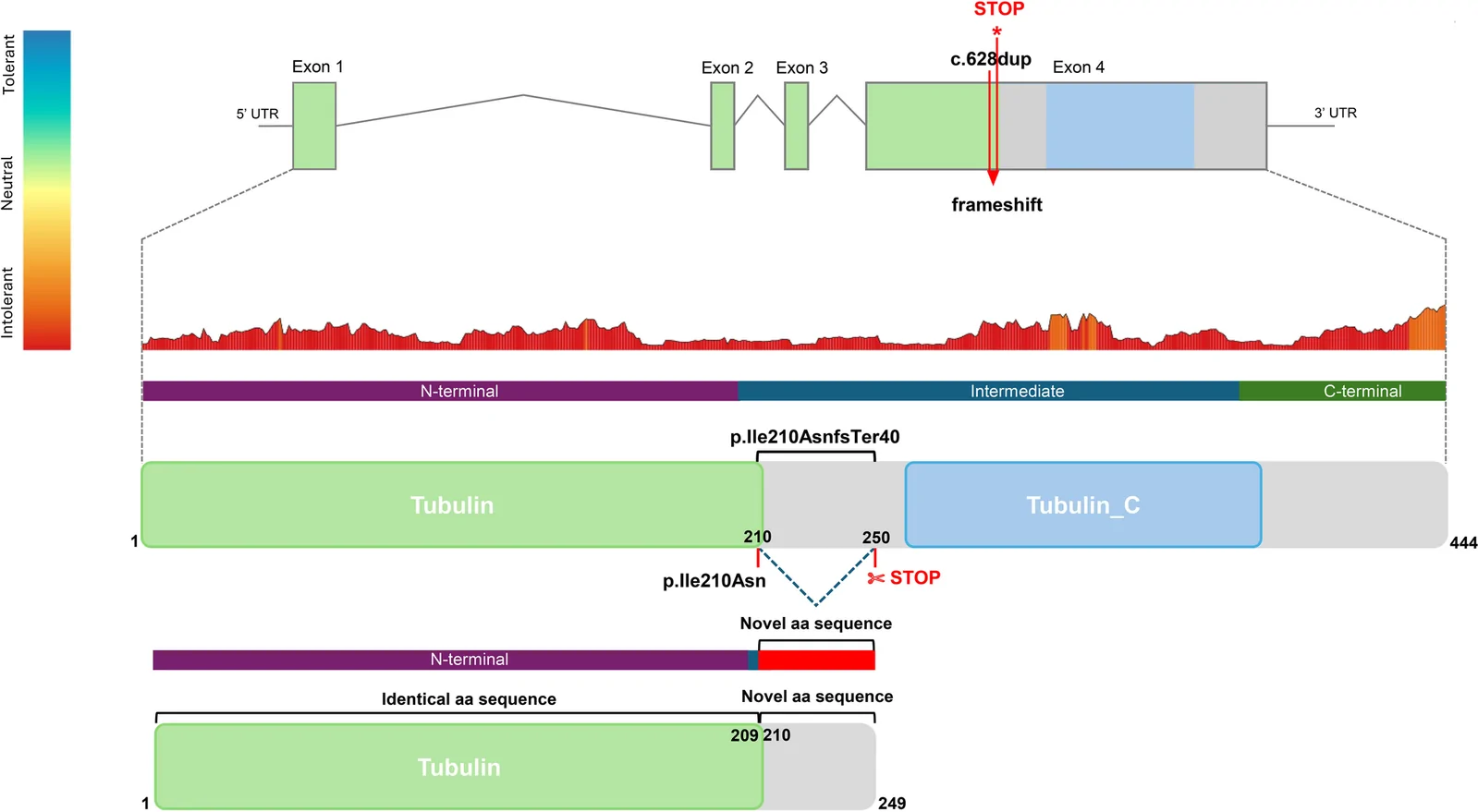
Schematic representation of TUBB gene and protein structure and position of the p.Ile210AsnfsTer40 mutation. Schematic representation of TUBB gene and protein structure illustrating the frameshift variant p.Ile210AsnfsTer40, resulting from the c.628dup variant. The c.628dup variant, located in exon 4, causes a frameshift leading to the introduction of a premature termination codon. At the protein level, the mutation causes the substitution of isoleucine in position 210 with asparagine, followed by a novel 40–amino acid frameshift-derived sequence and premature termination at residue 250. This results in loss of the C-terminal region and nearly all the intermediate domain of the tubulin protein. The upper panel shows the exon–intron organization of the *TUBB* gene, with the position of the variant located in the exon 4. MetaDome 2.0 tolerance landscape analysis highlights reduced mutational tolerance in the affected region. The schematic representation of TUBB protein depicts the N-terminal, intermediate, and C-terminal domains, alongside an independent Pfam-based domain annotation highlighting the Tubulin (PF00091, green) and Tubulin_C (PF03953, light blue) domains. Non-annotated regions are shown in grey. The exon colour scheme reflects the corresponding protein regions, illustrating gene–protein correspondence. The lower panel shows the predicted truncated protein product resulting from the mutation

The c.628dup mutation, located in exon 4 of *TUBB*, causes a frameshift that results in the introduction of a premature stop codon. At the protein level, the mutation leads to the substitution of isoleucine 210 with asparagine, an altered amino acid sequence from residues 210 to 249, and the loss of the normal downstream sequence due to early termination. This is expected to result in the absence of the entire C-terminal domain and nearly all the intermediate domain, ultimately producing a truncated protein of 249 amino acids which retains the wild-type *TUBB* sequence for the first 209 amino acids, followed by an aberrant 40–amino acid terminal sequence. Considering the predicted severe structural disruption of the protein, the well-established association of *TUBB* with neurodevelopmental disorders [2] and the recent association with macrothrombocytopaenia [27], the c.628dup variant was investigated to assess its pathogenic impact. Figure 2 shows a schematic representation of the TUBB gene and protein structure and the location of the p.Ile210AsnfsTer40 mutation.

### The p.Ile210AsnfsTer40 mutation affects TUBB transcript and protein stability and triggers TUBB proteasome-dependent degradation

To analyse the functional impact of p.Ile210AsnfsTer40 mutation, WT and mutant N-terminally MYC-tagged TUBB constructs were transfected into HEK-293 and COS-1 cells, and their protein and mRNA levels were evaluated by immunoblotting and qRT-PCR, respectively.

**Fig. 3:**
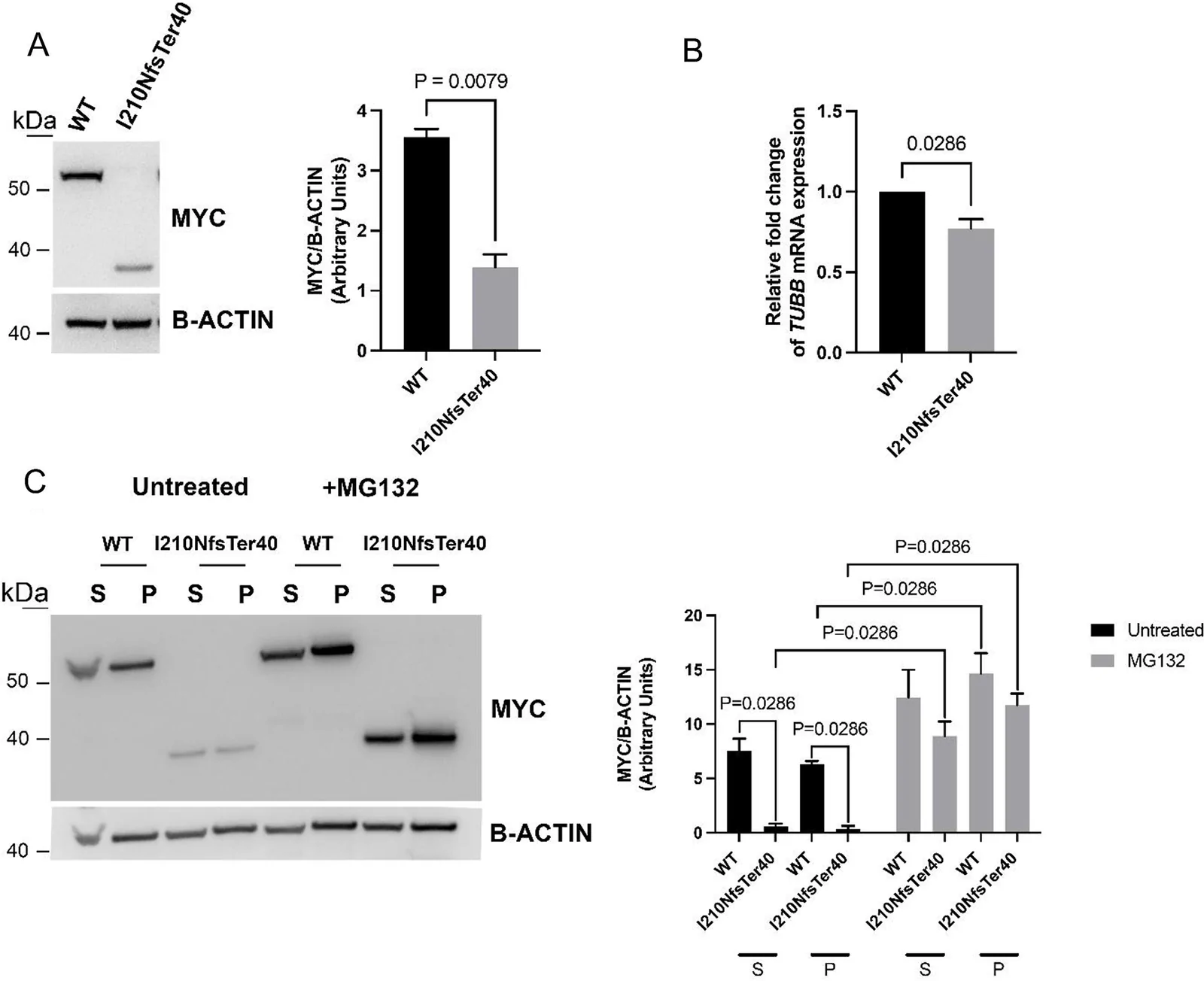
The p.Ile210AsnfsTer40 mutation affects TUBB mRNA and protein stability and triggers TUBB proteasome-dependent degradation. (**A**) Immunoblot analysis of HEK-293 cells transiently transfected with MYC-tagged TUBB (WT and mutant) documented a significant reduction of mutant tubulin. β-actin was used as loading control. The graph reports the mean ± SEM. *N* = 5, Mann Whitney test. (**B**) Gene expression of *TUBB* in transiently transfected HEK-293 cells detected by qRT-PCR documented a slight decrease in mutant *TUBB* mRNA levels. Expression values were normalized to *PGK1* expression. Histogram shows the quantitative analysis of *TUBB* gene expression. The graph reports the mean ± SEM. *N* = 5, Mann Whitney test. (**C**) Immunoblot analysis of HEK-293 cells over-expressing WT and mutated MYC-tagged TUBB and treated for 8 h with the proteasome inhibitor MG132 or with DMSO (untreated), revealed that the block of the proteasome system increases the accumulation of mutated TUBB, in a faster mode compared to WT, without changing its distribution from the detergent-soluble (S) to the detergent-insoluble (P) fraction. β-actin was used as loading control. The graph reports the mean ± SEM. *N* = 4, Mann Whitney test

Immunoblot analysis of transfected cells detected a truncated isoform of tubulin of approximately 35 kDa, whose protein levels were drastically reduced (Fig. 3A-Figure S2A).

To exclude that the reduction of mutant TUBB was due to a low transfection efficiency of its expression construct, HEK-293 cells were co-transfected with WT and mutant TUBB recombinant proteins and with the same amount of green fluorescent protein (GFP), which was used to assess the transfection rate. As shown in Figure S2B, while the protein levels of GFP were almost the same between HEK-293 cells over-expressing WT and mutant TUBB, mutant tubulin levels were specifically reduced.

Consistent with the reduced steady-state protein abundance, CHX-chase experiments showed an increased degradation kinetics of the mutant TUBB (Figure S3A). TUBB protein stability was additionally assessed by CHX-chase assays following proteasome inhibition, to allow a more reliable comparison between WT and mutant protein level, before CHX treatment. Notably, the truncated tubulin remained markedly unstable also after combined MG132/CHX treatment (Figure S3B).

We then evaluated the mRNA levels of WT and mutated *TUBB* in transfected HEK-293 cells. qRT-PCR experiments revealed a weak but significant reduction of the mutant transcript, thus indicating that c.628dup slightly impairs *TUBB* mRNA stability (Fig. 3B). Consistently, gene expression analysis performed in patient-derived fibroblasts confirmed a reduction of the mutant transcript (Figure S4), further supporting the decreased stability of *TUBB* mRNA harbouring the c.628 dup mutation.

We further investigated the contribution of the ubiquitin proteasome system, the major proteolytic intracellular pathway, to mutant TUBB turnover. For this reason, HEK-293 cells over-expressing WT and mutated TUBB were treated with the proteasome inhibitor MG132, and their detergent-soluble and insoluble protein fractions were analysed by western blotting.

As shown in Fig. 3C, total protein levels of the truncated TUBB were significantly increased after proteasome inhibition, as compared to those of WT tubulin, thus indicating that the mutant tubulin is efficiently disposed within the cell. Of note, after MG132 administration, WT and mutant TUBB were detected in both soluble and insoluble protein fraction, without observing significant differences in their distributions between the two portions (Fig. 3C).

### The p.Ile210AsnfsTer40 mutation affects TUBB interaction with α-tubulin and KIF1A motor protein and impairs TUBB localization

**Fig. 4:**
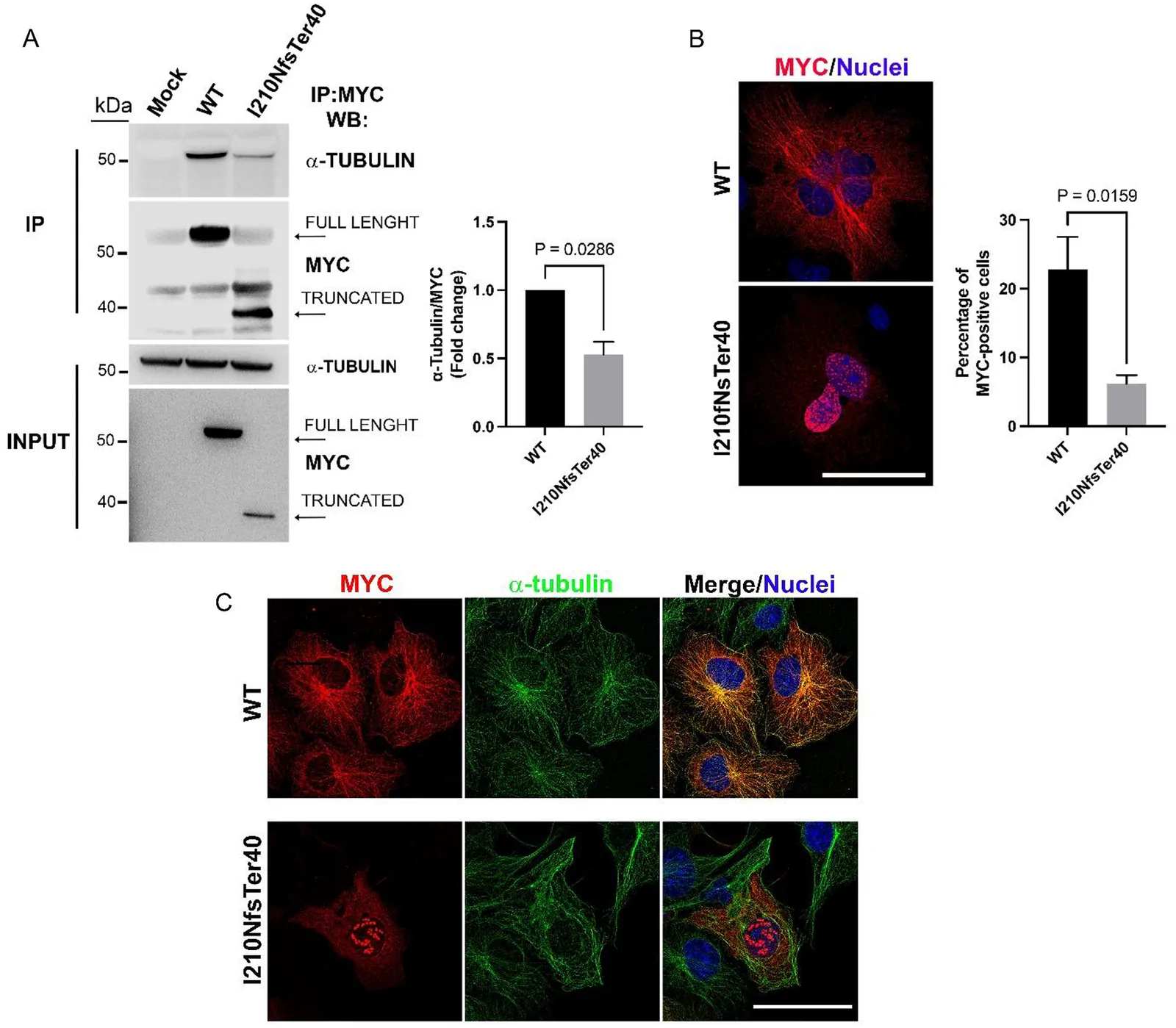
The p.Ile210AsnfsTer40 mutation affects TUBB-α-tubulin interaction and TUBB incorporation into microtubules, without affecting α-tubulin organization. (**A**) Co-immunoprecipitation assay was used to detect the interaction between the endogenous α-tubulin and recombinant TUBB (WT and mutant). The histogram shows the densitometric quantification of α-tubulin retained by each TUBB protein variant. The graph reports the mean ± SEM. *N* = 4, Mann Whitney test. (**B**) Immunocytochemical analysis of COS-1 cells over-expressing MYC-tagged TUBB (WT and mutant) showed that the WT tubulin is incorporated into microtubules while the mutated tubulin appears partially dispersed into cytoplasm and predominantly accumulates in the nucleus. Images were acquired using a 60× objective. Scale bar: 50 μm. The histogram shows the percentage of MYC-positive COS-1 cells over-expressing WT and mutated TUBB, calculated as: (Number of MYC positive cells/Number of total cell) x 100. The graph reports the mean ± SEM (848 cells from WT and 1322 cells from p.Ile210AsnfsTer40). Data were analysed by Mann Whitney test. (**C**) Immunocytochemical analysis of COS-1 cells overexpressing MYC-tagged TUBB (WT and mutant) labelled for MYC (red) and α-tubulin (green) showed no detectable differences in α-tubulin distribution between conditions. Images were acquired using a 60× objective. Scale bar: 50 μm

Since the p.Ile210AsnfsTer40 introduces a premature stop codon that causes the nearly and total loss of the intermediate and carboxy-terminal domains of TUBB respectively, we hypothesized that the truncated protein could display impaired heterodimer formation and interaction with microtubule-associated proteins. To test this hypothesis, we first evaluated the ability of mutant TUBB to interact with α-tubulin, which is essential for the formation of the α/β tubulin heterodimer. For this purpose, we performed co-immunoprecipitation assays on protein lysates obtained from HEK-293 cells over-expressing WT and mutant TUBB. As shown in Fig. 4A, while WT tubulin efficiently binds endogenous α-tubulin, TUBB mutant only partially retains this interaction, thus indicating that the lack of the intermediate and carboxy-terminal domains disrupts TUBB affinity for this protein, finally impairing the formation of functional tubulin heterodimers.

The cellular localization of mutated TUBB was then evaluated in transiently transfected cells.

According to co-immunoprecipitation experiments, immunocytochemical analysis revealed that TUBB mutant is not incorporated into the microtubules, with only a minor fraction appearing dispersed in the cytoplasm (Fig. 4B). Interestingly, immunofluorescence assays also showed a prominent nuclear localization of the truncated tubulin (Fig. 4B, Figure S5A). Consistent with the reduced stability and the proteasome dependent degradation of the mutant tubulin, the percentage of cells over-expressing the mutated TUBB and positive for the MYC-staining, was significantly lower as compared to that over-expressing the WT, thus confirming that the truncated variant is effectively degraded in a considerable percentage of cells (Fig. 4B, Figure S5B), which therefore result negative for the MYC-staining.

In addition, immunofluorescence analyses of endogenous α-tubulin in cells overexpressing mutant TUBB did not reveal evident alterations in α-tubulin localization and organization (Fig. 4C). These findings suggest the absence of a detectable dominant-negative effect of the mutant tubulin on microtubule organization.

**Fig. 5:**
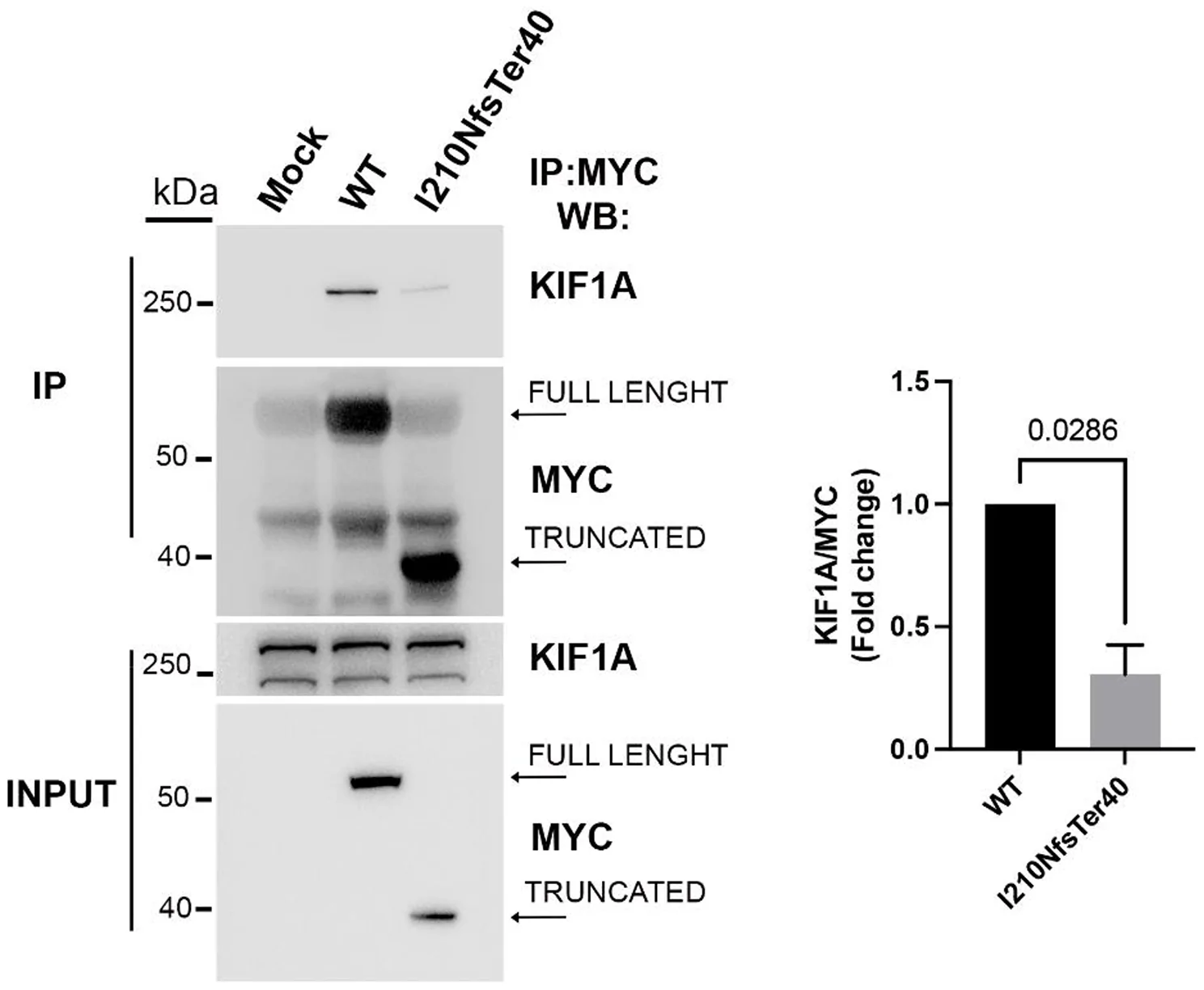
The p.Ile210AsnfsTer mutation affect TUBB interaction with KIF1A. Co-immunoprecipitation assay was used to detect the interaction between the endogenous KIF1A and recombinant TUBB (WT and mutant). The histogram shows the densitometric quantification of the endogenous KIF1A retained by each TUBB protein variant. The graph reports the mean ± SEM. *N* = 4, Mann Whitney test

We next assessed the impact of the p.Ile210AsnfsTer40 mutation on microtubule associated protein (MAP) binding by analysing, through co-immunoprecipitation experiments, the in vitro association between TUBB and the kinesin KIF1A. As shown in Fig. 5, while endogenous KIF1A co-immunoprecipitated with WT recombinant tubulin, this interaction is almost completely disrupted for the TUBB mutant, thus indicating that the p. Ile210AsnfsTer40 impedes the binding between TUBB with this motor protein.

### The p.Asp249Val substitution impairs TUBB protein stability and affinity for α-tubulin, and affects TUBB subcellular localization

Recently, the p.Asp249Val heterozygous substitution in *TUBB* has been reported to induce TUBB nuclear localization, affecting microtubule assembly and resulting in inherited bone marrow failure with macrothrombocytopaenia [27].

Considering the similarity of haematological phenotypes caused by the p.Asp249Val and p.Ile210AsnfsTer40 mutations, we decided to deeper investigate the pathogenic effects of the p.Asp249Val substitution.

For this reason, we introduced the p.Asp249Val pathogenic variant into the WT MYC-tagged TUBB construct, and the expression vector was used to transiently transfect HEK-293 and COS-1 cells.

**Fig. 6:**
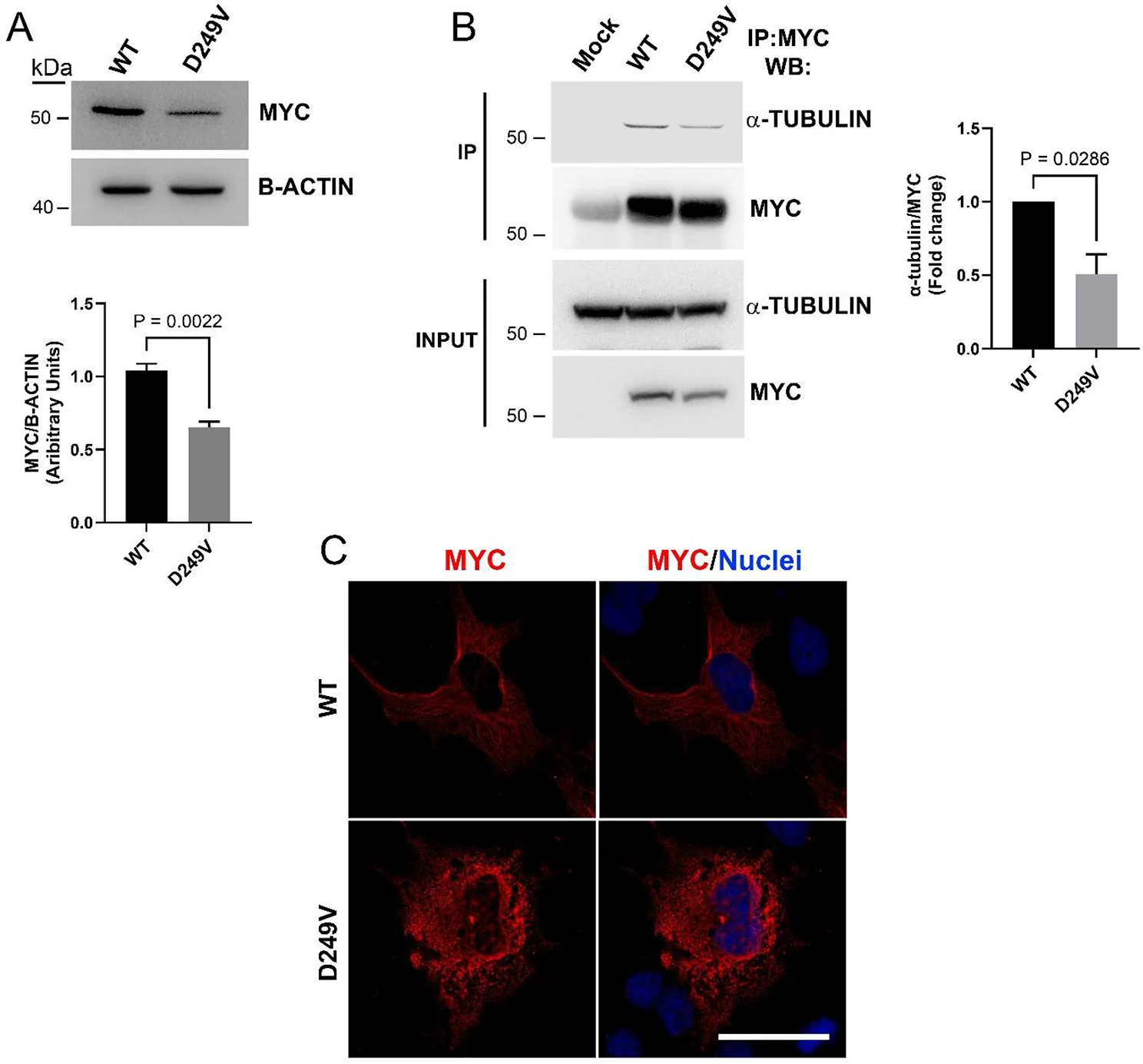
The p.Asp249Val substitution affects TUBB stability and interaction with α-tubulin and impairs TUBB subcellular localization. (**A**) Immunoblot analysis of HEK-293 cells transiently transfected with expression constructs encoding for MYC tagged TUBB (WT and mutant) documented a slight but significant reduction of the mutant tubulin. β-actin was used as loading control. The graph reports the mean ± SEM. *N* = 5, Mann Whitney test. (**B**) Co-immunoprecipitation assay was used to detect the interaction between the endogenous α-tubulin and recombinant TUBB (WT and mutant). Histograms show the densitometric quantification of α-tubulin retained by each TUBB protein variant. The graph reports the mean ± SEM. *N* = 4, Mann Whitney test. (**C**) Immunocytochemical analysis of COS-1 cells over-expressing MYC-tagged TUBB (WT and mutant) showed that the WT tubulin is correctly incorporated into microtubules while the mutated tubulin appears mainly dispersed into the cytoplasm and displays a more marked nuclear localization. Images were acquired using a 60× objective. Scale bar: 50 μm

We first evaluated the protein levels of the TUBB mutant. Immunoblot analysis conducted on transfected HEK-293 cells documented a moderate reduction of mutated tubulin, thus suggesting that the p.Asp249Val substitution may affect its short-term protein stability (Fig. 6A).

To further prove the effect of the p.Asp249Val mutation on TUBB protein stability, protein levels of WT and mutated TUBB were monitored in transiently transfected HEK-293 cells, following treatment with the protein synthesis inhibitor CHX. Accordingly, immunoblot analysis suggested a faster decline of the mutant protein compared with WT TUBB (Figure S6).

Moreover, similarly to the p.Ile210AsnfsTer40 variant, MG132 treatment increased protein levels of mutant tubulin in HEK-293 cells over-expressing the recombinant tubulin harbouring the p. D249V substitution. In addition, no changes in its distribution between detergent-soluble and insoluble protein fractions were observed (Figure S7).

These findings support a contribution of the proteasome-mediated degradation to the reduced steady-state abundance of the mutant protein.

Since the p.Asp249Val mutation affects a highly conserved residue (Figure S8) in the GTP-binding site, required for intradimer interface and α/β tubulin heterodimer formation, we evaluated TUBB interaction with the endogenous α-tubulin. Co-immunoprecipitation experiments showed that the p.Asp249Val substitution slightly affects TUBB binding for α-tubulin, thus suggesting that this mutation may reduce the proper tubulin heterodimer formation (Fig. 6B).

**Fig. 7:**
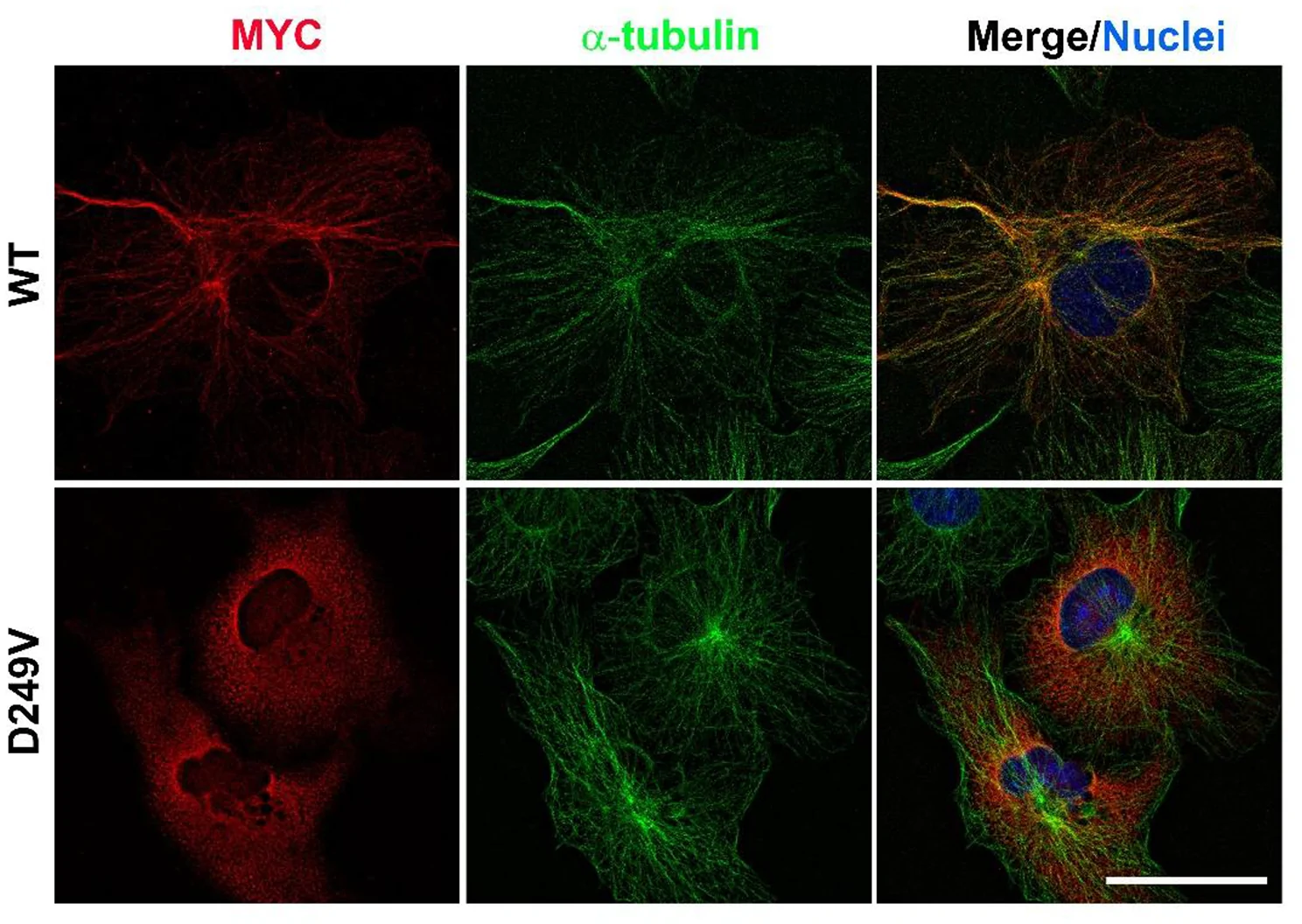
The p.Asp249Val mutation in TUBB does not affect α-tubulin localization and organization. Immunocytochemical analysis of COS-1 cells overexpressing MYC-tagged TUBB (WT and mutant) labelled for MYC (red) and α-tubulin (green) showed no detectable differences in α-tubulin distribution between conditions. Images were acquired using a 60× objective. Scale bar: 50 μm

We then explored the effect of the p.Asp249Val pathogenic variant on microtubule architecture in COS-1 cells. Accordingly with Shah and colleagues [27], immunocytochemical analysis revealed a more marked nuclear staining of the mutant tubulin (Fig. 6C). In addition, as previously observed for the TUBB harbouring the p.Ile210AsnfsTer40 truncated mutation, immunofluorescence analysis showed that the mutant tubulin is depleted from microtubules, mainly appearing as dispersed among the cytoplasm (Fig. 6C). Similarly to the p.Ile210AsnfsTer40 variant, confocal analysis of COS-1 cells overexpressing the p.Asp249Val mutant did not reveal significant alterations in α-tubulin organization (Fig. 7), indicating that neither mutant protein induces detectable disruption of the endogenous microtubule network.

## Discussion

Mutations in tubulin genes cause a wide range of pathological conditions collectively known as tubulinopathies and historically considered a broad class of congenital and non-progressive disorders of brain neurodevelopment [28, 29]. Nevertheless, in the last 20 years, an increasing number of tubulin mutations have also been linked to neurodegenerative diseases [7–13] and non-neurological conditions [14–27], thus necessitating a broader redefinition of their phenotypic spectrum. 

*TUBB* encodes for a β-tubulin isotype highly expressed in human foetal brain [2], mice developing skin [30] and human fibroblasts [31]. Moreover, its expression has been detected in neonatal platelets [32] and haematopoietic progenitors, including megakaryocyte and erythroid progenitors [33].

Mutations in *TUBB* have been associated with a wide range of clinical conditions including brain malformations, skin creases and, more recently, macrothrombocytopaenia [2, 5, 27, 30, 31].

Here, we report on a novel heterozygous truncating *TUBB* mutation (c.628dup; p.Ile210AsnfsTer40), consisting of a frameshift variant that leads to the premature termination of tubulin synthesis, determining the loss of the intermediate and carboxy-terminal domains in the truncated TUBB, and identified in a subject affected by thrombocytopaenia, anaemia and neurological impairment.

We investigated the pathogenic effect of the present disease-related mutation and identified a combined defect of protein stability and functions.

Immunoblot analysis of cells over-expressing mutant TUBB revealed a markedly reduced truncated protein isoform. In addition, gene expression analyses in transfected HEK-293 cells and in patient-derived fibroblasts revealed a modest but significant reduction of the mutant transcript, suggesting that the c.628dup variant may affect *TUBB* mRNA stability.

We also demonstrated that the p.Ile210AsnfsTer40 mutation triggers the proteasome-dependent degradation of TUBB, since proteasome inhibition strongly increased its protein levels. Moreover, we observed that the proteasome block does not induce, as previously reported for the *TUBA1A* haploinsufficiency conditions associated with neurodegeneration in humans and mice [13, 34], the selective accumulation of the unstable tubulin in the detergent-insoluble protein fraction.

These findings suggest distinct proteostasis behaviours between mutant TUBB and TUBA1A, which may contribute, at least in part, to the divergent phenotypic outcomes.

The p.Ile210AsnfsTer40 frameshift variant introduces a premature stop codon at amino acid position 250, resulting in a truncated tubulin lacking most of the intermediate domain and the entire carboxy-terminal region, which are required for tubulin heterodimer formation and molecular motor binding, respectively. Based on the predicted loss of these domains, both interactions were expected to be severely impaired, with the loss of the carboxy-terminal region suggesting a complete disruption of interactions with molecular motors. To validate these predictions in a cellular context, we performed co-immunoprecipitation experiments which revealed differential effects of the p.Ile210AnsfsTer40 on these binding. Indeed, the mutant protein retained a partial association with α-tubulin whereas interaction with the molecular motor KIF1A was markedly reduced, although not completely abolished. The residual TUBB–α-tubulin and TUBB-KIF1A associations may reflect indirect interactions mediated by additional proteins that co-immunoprecipitated with mutant TUBB than preserved direct binding.

According to the impaired in vitro association between the mutated TUBB and α-tubulin, immunocytochemical analysis showed that the shortened tubulin was not incorporated into microtubules, with a minor fraction appearing diffusely distributed throughout the cytoplasm. Moreover, its reduced steady-state detectability in MYC-positive transfected cells, further supports its rapid degradation and loss of stable incorporation into microtubules.

Interestingly, immunofluorescence assays also showed an aberrant nuclear localization of the mutated TUBB. This mis-localization may reflect a passive redistribution of the truncated tubulin, likely facilitated by its reduced molecular size, that escapes proteasome-mediated degradation.

The accumulation of TUBB at nuclear level has been recently reported to be caused by the p.Asp249Val substitution, identified in a subject affected by macrothrombocytopaenia [27].

Similarly to the p.Ile210AsnfsTer40 mutation, we demonstrated that the p.Asp249Val substitution affects TUBB protein stability and association with α-tubulin, finally preventing the assembly of TUBB into microtubules.

Moreover, immunocytochemical analyses showed that both mutant tubulins do not alter α-tubulin staining, suggesting that p.Ile210AsnfsTer40 and p.Asp249Val mutations do not exert a dominant-negative effect on endogenous microtubule organization.

Overall, this evidence argues for an overlapping of pathogenic mechanisms for p.Ile210NfsTer40 and p.Asp249Val mutations and suggest that TUBB loss functions and stability may contribute to cause thrombocytopaenia in humans.

It has been widely proved that megakaryocyte maturation and proplatelet formation depend on the strict regulation of microtubules. These cytoskeletal structures organize into mitotic spindles to mediate megakaryocyte polyploidization and provide forces for megakaryocyte cytoplasm remodelling into proplatelet extensions [35]. Furthermore, microtubules arrangement into bipolar arrays in proplatelets allows the transport of organelles from megakaryocyte body [36], which is mediated by kinesins. Microtubules also maintain discoid shape of platelets and drive, during platelet activation, structural transitions from discoid to a more spherical platelet shape [37].

Thus, since *TUBB* expression has been detected in both megakaryocytes and neonatal platelets [32, 33] we hypothesize that the p.Ile210AsnfsTer40 pathogenic variant may impair platelet biogenesis and functions, by reducing the microtubule content in both megakaryocyte and platelets and affecting microtubule-dependent transport in megakaryocytes.

We further propose that nuclear redistribution of the truncated protein may represent an additional mechanism potentially affecting megakaryocyte maturation. Although speculative, TUBB mis-localization may interfere with endomitotic processes required for megakaryocyte polyploidization. Therefore, although primary pathogenic mechanism appears to involve loss of protein stability and functions, the mutant tubulin may also exert secondary effects on megakaryocyte maturation through its aberrant sub-cellular localization.

Compelling evidence demonstrates a pivotal role of the proteasome in regulating platelet thrombopoiesis and lifespan. Indeed, proteasome inhibition has been demonstrated to reduce platelet count and half-life, causing thrombocytopaenia in patients treated with the proteasome inhibitor Bortezomib [38]. Moreover, mice deficient for the PSMC1 subunit of the proteasome in platelets, exhibited severe thrombocytopaenia [39]. In addition, in ANKRD26-related thrombocytopenia, cytoplasmic particulate structures enriched in proteasome components and polyubiquitinated proteins have been described [40], further supporting the involvement of proteostatic pathways in platelet biology. Consistently, platelet activation is associated with increased proteasomal activity [41], while loss-of-function mutations in genes encoding proteasome subunits [42], are frequently associated with thrombocytopaenia.

Thus, since we have demonstrated that the p.Ile210AsnfsTer40 variant triggers proteasome-dependent degradation of TUBB, it is intriguing to hypothesize that the mutant protein may impose, overtime, a sustained proteostatic burden in megakaryocytes and platelets, compromising platelet production and lifespan, and contributing to the observed haematological phenotype.

Although a combined mechanism involving both a peripheral immune-mediated thrombocytopenia and a central marrow defect cannot be excluded in the patient harbouring the p.Ile210AsnfsTer40 mutation, several clinical and biological findings are consistent with an underlying bone marrow defect.

Indeed, the patient showed an initial biphasic haematological presentation characterized by an early transient trilineage cytopenia, followed by persistent and progressively worsening thrombocytopenia. Notably, bone marrow evaluation from the very first evaluation showed marked hypo-dysmegakaryocytopoiesis and reduced cellularity for age (~ 60%), performed at a time when thrombocytopenia was still mild supporting an intrinsic defect in megakaryocyte development rather than a secondary immune-mediated process.

This interpretation is further supported by the absence of specific anti-platelet antibodies at first evaluation despite established thrombocytopenia, and by their subsequent intermittent positivity over time, which is more consistent with an epiphenomenon than with a primary pathogenic driver. In addition, the lack of response to immunosuppressive therapy and thrombopoietin receptor agonists argues against a predominantly immune-mediated thrombocytopenia.

Taken together, these findings support the hypothesis that the cytopenias primarily originate from an intrinsic bone marrow defect, although a concomitant immune-mediated component contributing to thrombocytopenia cannot be completely ruled out. Moreover, although platelet function studies were not performed, the absence of clinically relevant bleeding manifestations may indirect evidence suggests relatively preserved platelet function.

Finally, the heterozygous *HBB* variant inherited from the mother and consistent with β-thalassemia carrier status, is unlikely to explain the early normocytic hyporegenerative anaemia, as a single heterozygous mutation is insufficient to impair haemoglobin production or erythroid maturation. Moreover, a normal mean corpuscular volume finding is rarely present in β-thalassemia carrier and described in association with some mild variants.

Lastly, *TUBB* is highly expressed in the developing brain and plays a key role in neurogenesis, and its disruption has been associated with structural brain abnormalities [2]. Recent evidence has further linked *TUBB* mutations to impaired primary ciliogenesis and cerebellar involvement, highlighting the functional connection between microtubules, primary cilia, and cerebellar development [43].

Indeed, it has been demonstrated that a subset of *TUBB* mutations impairs early ciliogenesis by affecting microtubule nucleation, growth, and stability, as well as dynein-dependent trafficking, which are essential for cilium assembly. The authors also showed that *TUBB* haploinsufficiency is not sufficient to impair ciliogenesis in engineered non neuronal haploid models, in which a moderate decrease of *TUBB* was reported, suggesting that its partial dosage reduction does not disrupt ciliary assembly [43]. However, it cannot be excluded that in neurons, in which *TUBB* is highly expressed during foetal brain development [2], its reduced dosage and functional availability may have more pronounced consequences on ciliogenesis. In this context, a potential contribution of the reduced TUBB transcript and protein levels associated with the p.Ile210AsnfsTer40 variant to ciliary dysfunction and cerebellar abnormalities cannot be ruled out. Neurons are also particularly sensitive to perturbations in proteostasis [44, 45]; as a result, impaired degradation of the truncated protein may burden the proteasomal system, potentially leading to its accumulation and indirectly affecting ciliogenesis through mechanisms not attributable to a reduced TUBB dosage.

These observations suggest that the cerebellar vermis dysplasia observed in our patient, may reflect disrupted TUBB-dependent cerebellar development, potentially arising from impaired early ciliogenesis and microtubule-dependent neuronal processes.

Finally, impaired interaction between TUBB and the neuron-specific kinesin KIF1A may represent an additional mechanism contributing to the neurological phenotype observed in our patient. Indeed, the kinetics of axonal transport critically depend on intact microtubule tracks and adequate microtubule density [46].

Since the p.Ile210AsnfsTer40 variant reduces both TUBB transcript and protein levels, and the small amount of truncated protein produced is unable to incorporate into the microtubule lattice and interact with KIF1A, we hypothesize that neurons expressing this variant may have insufficient TUBB availability to support proper microtubule assembly, limiting KIF1A-mediated cargo transport and reducing axonal transport efficiency.

Accordingly, KIF1A-related disorders share overlapping clinical and neuroimaging features with tubulinopathies, including cerebellar involvement and structural brain abnormalities [47, 48], suggesting a convergent pathogenic mechanism involving disrupted microtubule-dependent transport.

### Study limitations

Some limitations of the study must be considered. First, our experimental findings are based on a single affected individual, which precludes definitive genotype–phenotype correlations. Second, functional assays relied on heterologous overexpression systems, which may not fully recapitulate endogenous *TUBB* behaviours. Moreover, although patient-derived fibroblasts confirmed reduced *TUBB* transcript levels, the endogenous protein expression and subcellular localization of the truncated protein remain uncharacterized. Additional studies in relevant physiological systems, including patient-derived neuronal and blood-derived models, are warranted to further define the functional consequences of TUBB dysfunction.

## Conclusions

In conclusion, the present study identifies the novel heterozygous truncating p.Ile210AsnfsTer40 mutation in *TUBB* in a patient affected by a multisystem disorder characterized by haematological and neurological manifestations, further expanding the phenotypic spectrum of rare paediatric diseases related to this gene.

Our findings suggest that impaired TUBB protein stability, defective incorporation into microtubule and altered TUBB proteostasis and MAP interaction, represent key pathogenic mechanisms underlying this condition, supporting a model in which *TUBB* loss of function and stability may contribute to cause thrombocytopaenia in humans.

These findings further highlight the importance of β-tubulin in both neuronal and platelet biology, providing new insights into the molecular basis and clinical variability of tubulinopathies.

Moreover, our data also suggest that additional pathogenic mechanisms, such as proteostasis stress overtime and nuclear accumulation of mutant tubulin, may also contribute to disease pathogenesis.

## Supplementary Information

Below is the link to the electronic supplementary material.

Supplementary Material 1

Supplementary Material 2

## Data Availability

The datasets used and analysed during the current study are available from the corresponding author on reasonable request.

## Abbreviations

KIF1A: Kinesin family member 1 A
WT: Wild type
HEK-293: Human embryonic kidney 293
CHX: Cycloheximide
qRT-PCR: Quantitative Real-time PCR
5’ UTR: 5’ untranslated region
PGK1: Phosphoglycerate Kinase 1
MRI: Magnetic resonance imaging
GFP: Green fluorescent protein
MAP: Microtubule associated protein
