# Supplementary material for: Novel heterozygous truncating variant in TUBB associated with thrombocytopaenia and neurological abnormalities
Source: Orphanet J Rare Dis. 2026 Aug 13;21:415. doi: 10.1186/s13023-026-04546-5 (PMC13757095; doi:10.1186/s13023-026-04546-5)
Supplement: Supplementary file 1 [file 13023_2026_4546_MOESM1_ESM.pdf]

Novel heterozygous truncating variant in *TUBB* associated with thrombocytopaenia and neurological abnormalities
Svezia *et al*.

Supplementary Material

- **Supplementary Figure 1**: Visualization of aligned reads using the Integrative Genomics Viewer confirming the *de novo TUBB* c.628dup variant in the proband and its absence in parents **Supplementary Figure 2**: Protein levels of WT and mutant TUBB harboring the p.Ile210AsnfsTer40 pathogenic variant in transfected COS-1 and HEK-293 cells
- **Supplementary Figure 3**: Short-term protein stability of WT and mutant TUBB harboring the p.Ile210AsnfsTer40 mutation after CHX and combined MG132/CHX treatment in transfected HEK-293 cells
- **Supplementary Figure 4:** Gene expression levels of *TUBB* in control and patient-derived primary fibroblasts
- **Supplementary Figure 5**: Sub-cellular localization and transfection rate of WT and mutant TUBB harboring the p.Ile210AsnfsTer40 pathogenic variant in HEK-293 cells
- **Supplementary Figure 6**: Short-term protein stability of WT and mutant TUBB harboring the p.Asp249Val pathogenic variant in transfected HEK-293 cells
- **Supplementary Figure 7:** Immunoblot analysis of WT and mutant TUBB harboring the p.Asp249Val pathogenic variant after proteasome inhibition in HEK-293 cells
- **Supplementary Figure 8**: Multiple sequence alignment of β-tubulin isoforms affected by the substitution p.Asp249Val
- **Supplementary Table1:** List of primers used for qRT-PCR experiments in transfected HEK-293 cells
- **Supplementary Table2:** List of primers used for qRT-PCR experiments in primary fibroblasts
- **Supplementary Table 3:** Main haematological parameters of the patient over time
- **Supplementary Table 4:** Gene list for inherited anaemia and bone marrow failure included in the NGS panel

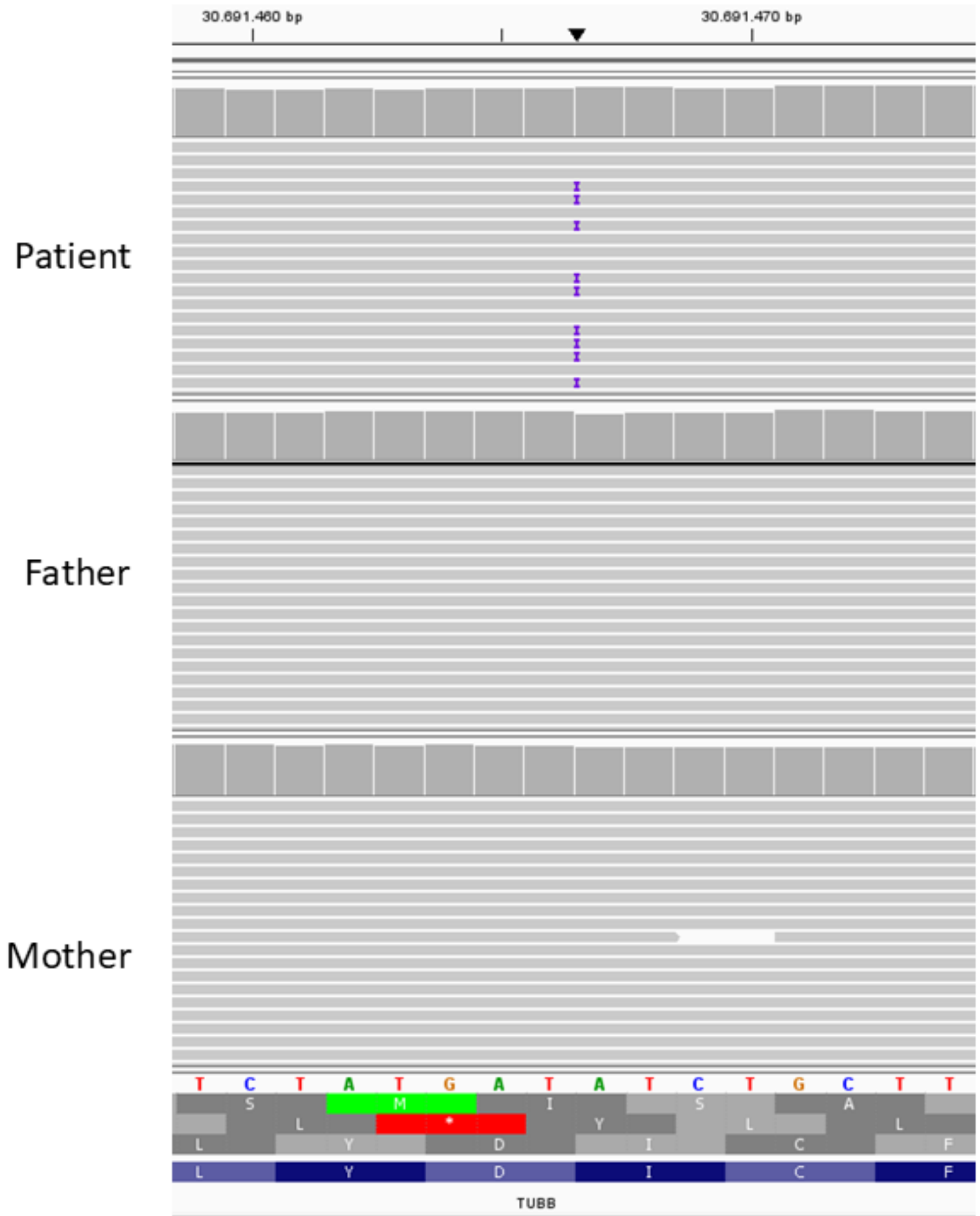

Supplementary Figure 1. Visualization of *de novo TUBB* c.628dup variant in proband and parents using Integrative Genomics Viewer.The variant is detected in heterozygous state in the proband (top), while both parents (middle, father; bottom, mother) show only reference reads at adequate coverage, confirming its *de novo* origin.

•

•

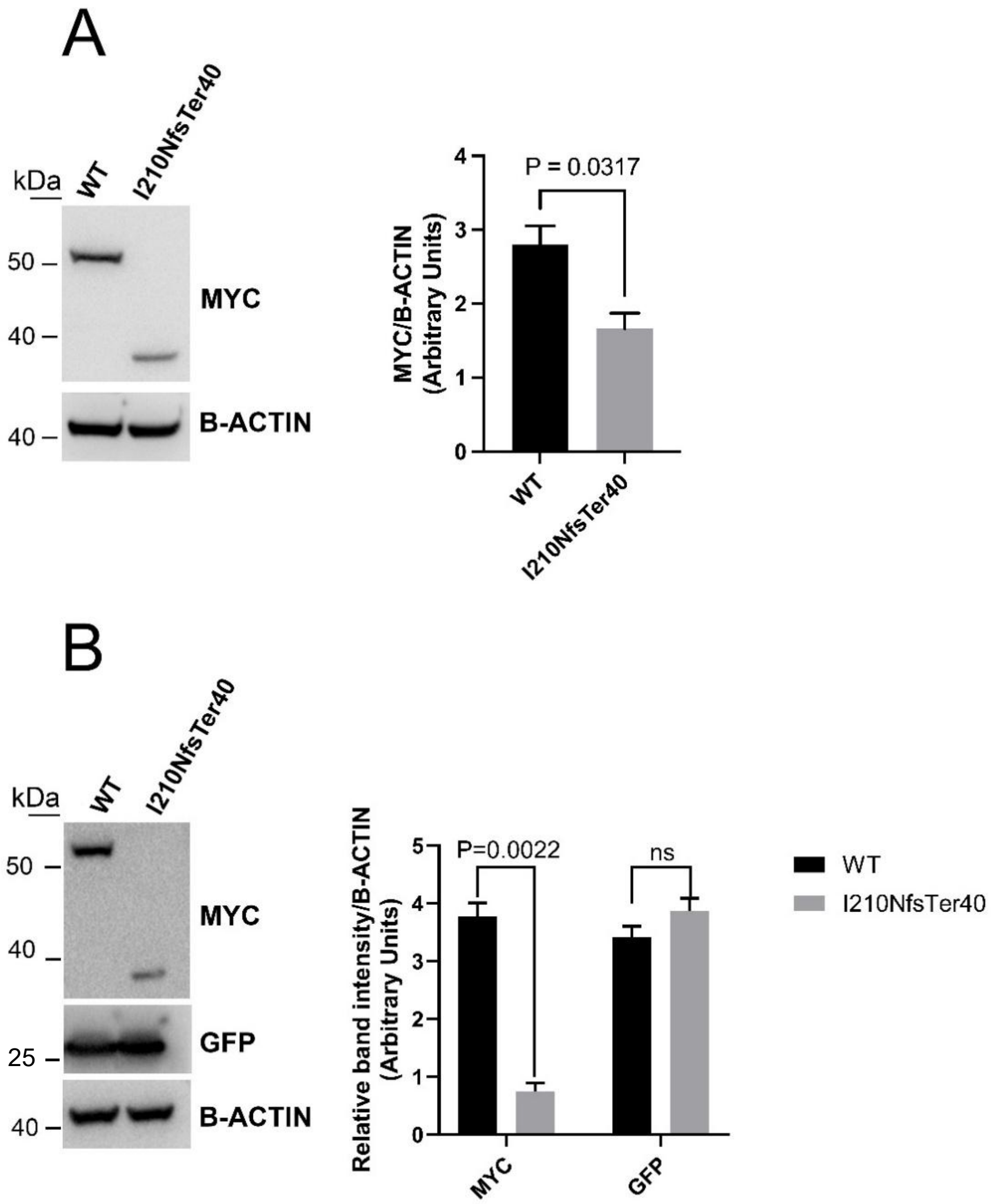

Supplementary Figure 2. The p.Ile210AsnfsTer40 mutation affects TUBB protein stability. (A) Immunoblot analysis of COS-1 cells transiently transfected with MYC-tagged TUBB (WT and mutant) documented a significant reduction of the mutant tubulin. β-actin was used as loading control. The graph reports the mean ± SEM. N= 5, Mann Whitney test. (B) Immunoblot analysis of HEK-293 cells co-transfected with the same amount (1μg) of TUBB (WT and mutant) and GFP constructs, showed that the protein levels of the mutated tubulin were specifically reduced. The graph reports the mean ± SEM. N=6, Mann Whitney test.

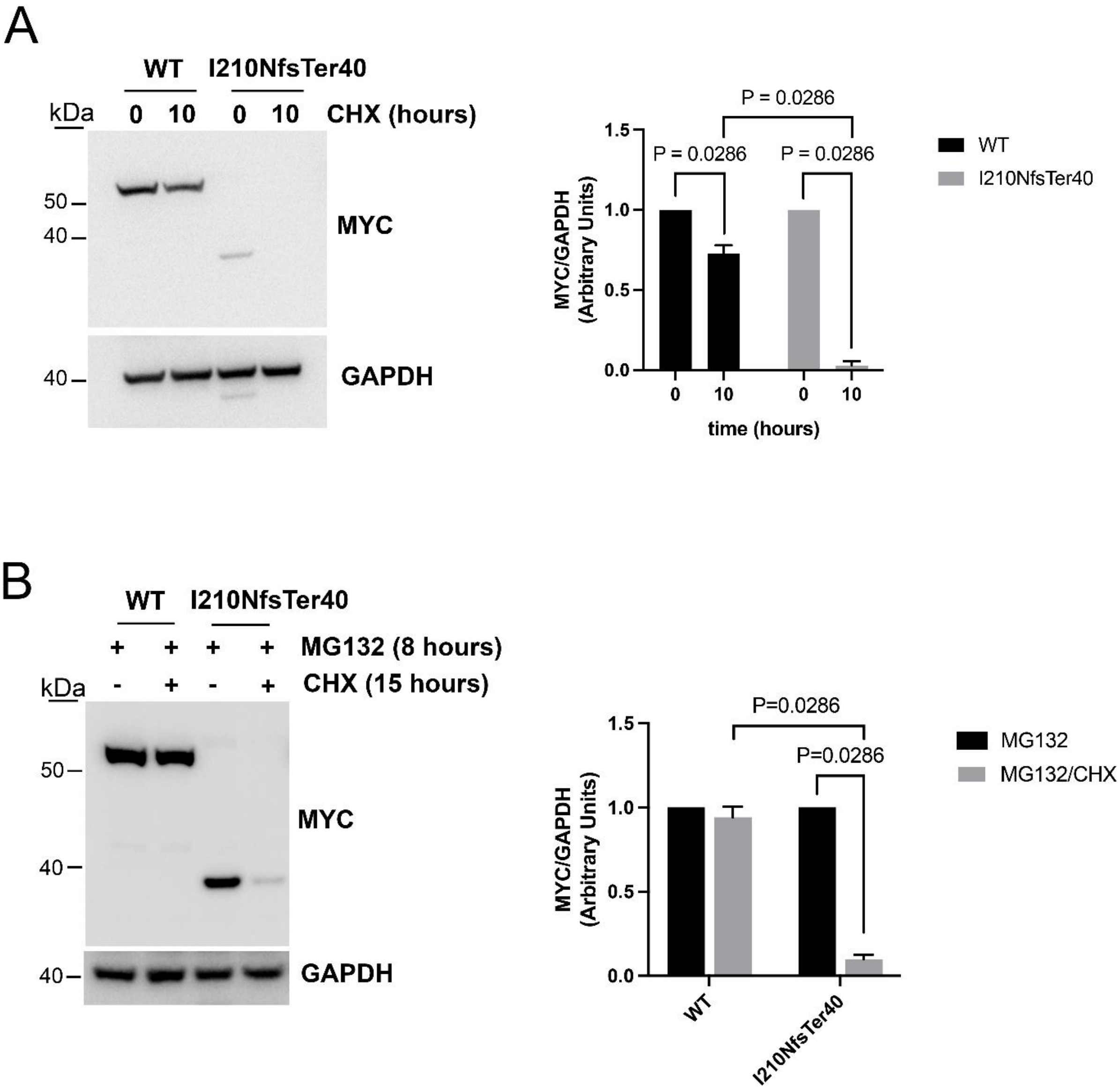

Supplementary Figure 3. The p.Ile210AsnfsTer40 mutation affects TUBB short-term protein stability. (A) Immunoblot analysis of HEK-293 cells transfected with the MYC-tagged TUBB (WT and mutant) before and after CHX treatment (10 hours). On the right a graphical representation of TUBB protein stability before (t0) and after (t10) CHX treatment. Protein levels were normalized to GAPDH and the relative protein intensities were compared to the 0 hour of WT or mutated TUBB. The graph reports the mean ± SEM. N=4, Mann Whitney test. (B) HEK-293 cells transiently transfected with the MYC-tagged TUBB (WT and mutant) were pre-treated with MG132 for 8 hours, to allow a more reliable comparison between WT and mutant protein level, followed by CHX treatment for 15 hours. TUBB protein levels were determined by western blotting after MG132 and combined MG132/CHX treatment. On the right, a graphical representation of TUBB protein stability. Protein levels were normalized to GAPDH and the relative protein intensities

were compared to MG132 treatment condition of WT or mutated TUBB. The graph reports the mean ± SEM. N=4, Mann Whitney test.

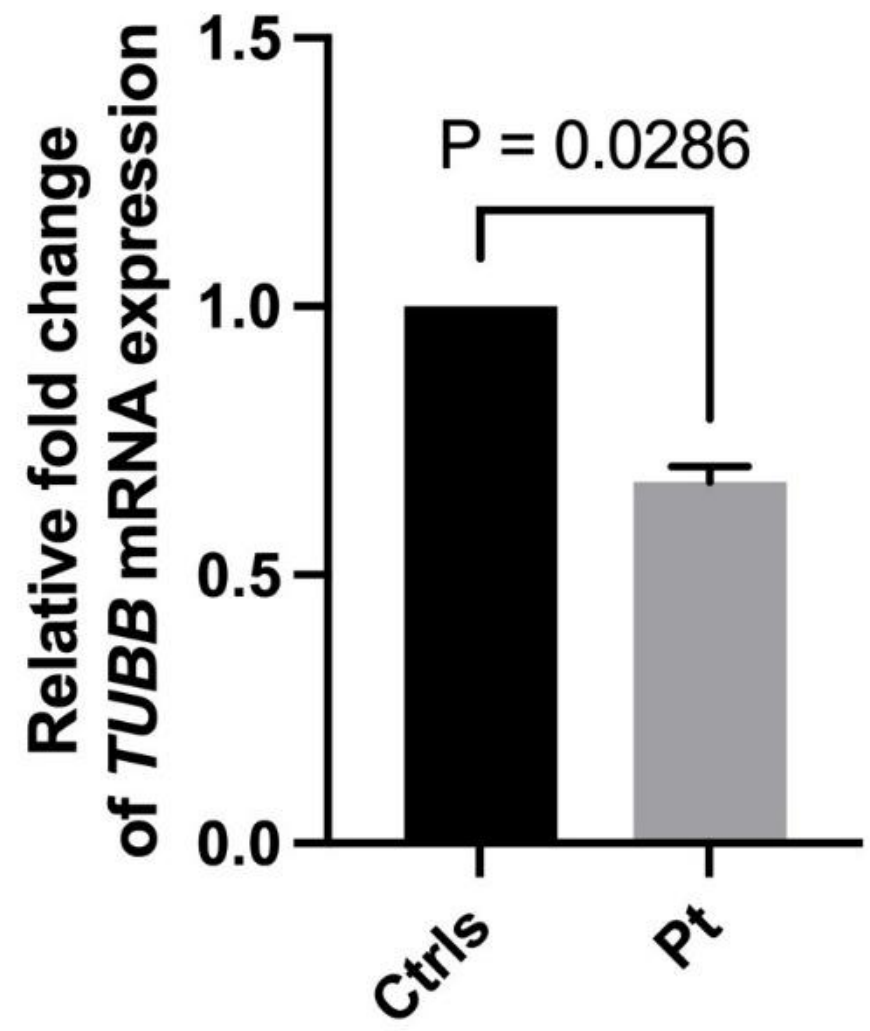

Supplementary Figure 4. The p.Ile210AsnfsTer40 mutation affects *TUBB* mRNA stability in primary fibroblasts. Gene expression of *TUBB* in controls and patient derived primary fibroblast, detected by qRT-PCR, documented a slight decrease in mutant *TUBB* mRNA levels. Expression values were normalized to *PGK1* expression. Histogram shows the quantitative analysis of *TUBB* gene expression. The graph reports the mean ± SEM. N=4, Mann Whitney test.

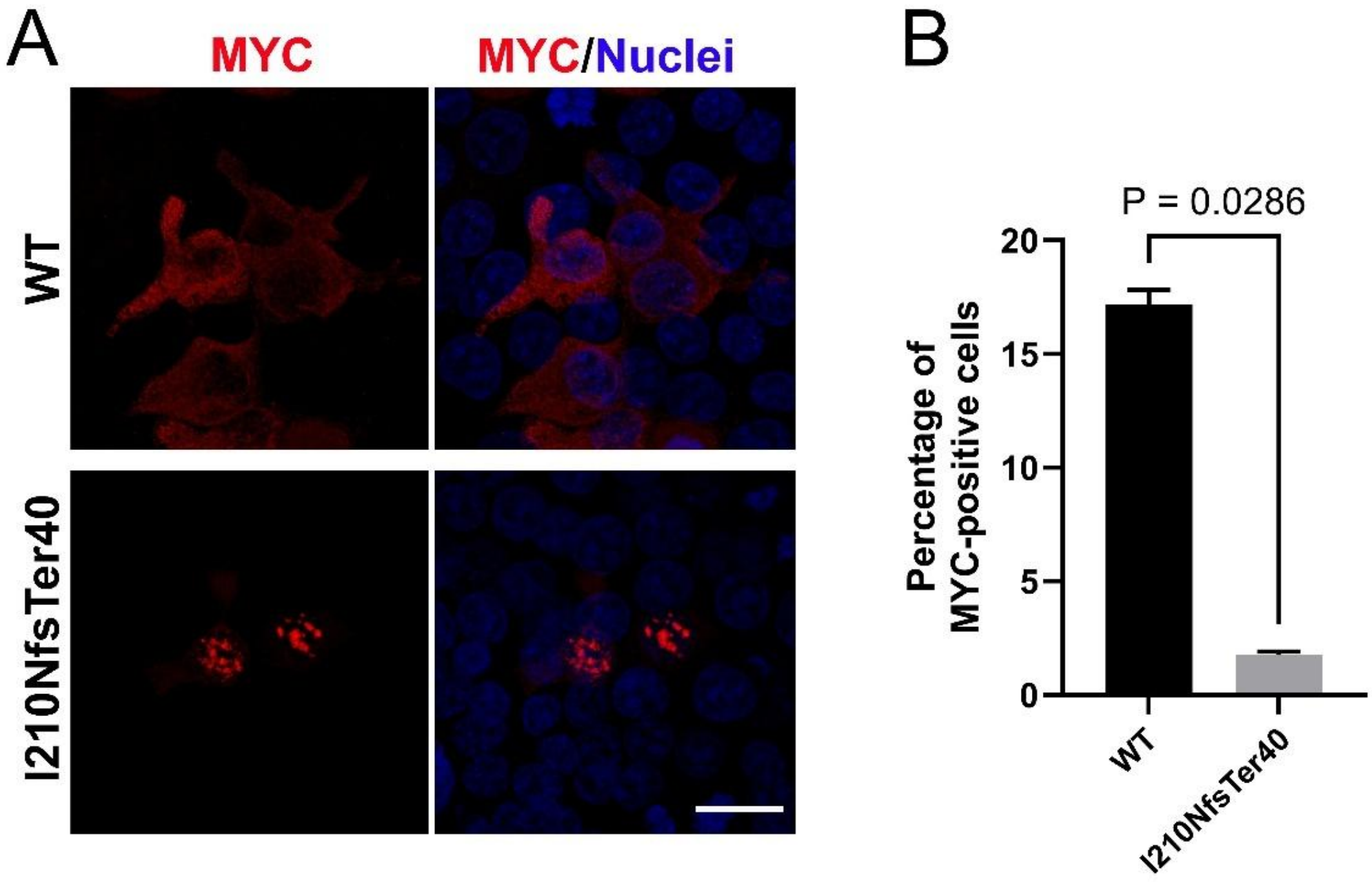

Supplementary Figure 5. The p.Ile210AsnfsTer40 mutation affects TUBB localization, promoting its accumulation in the nucleus. (A) Immunocytochemical analysis of HEK-293 cells over-expressing MYC-tagged TUBB (WT and mutant) showed that the mutated tubulin accumulates in the nucleus. Images were acquired using a 60× objective. Scale bar: 50 µm. (B) The histogram shows the percentage of MYC-positive HEK-293 cells over-expressing WT and mutated TUBB, calculated as: (Number of MYC positive cells/Number of total cell) x 100. The graph reports the mean ± SEM (1908 cells from WT and 2072 cells from p.Ile210AsnfsTer40). Data were analyzed by Mann Whitney test.

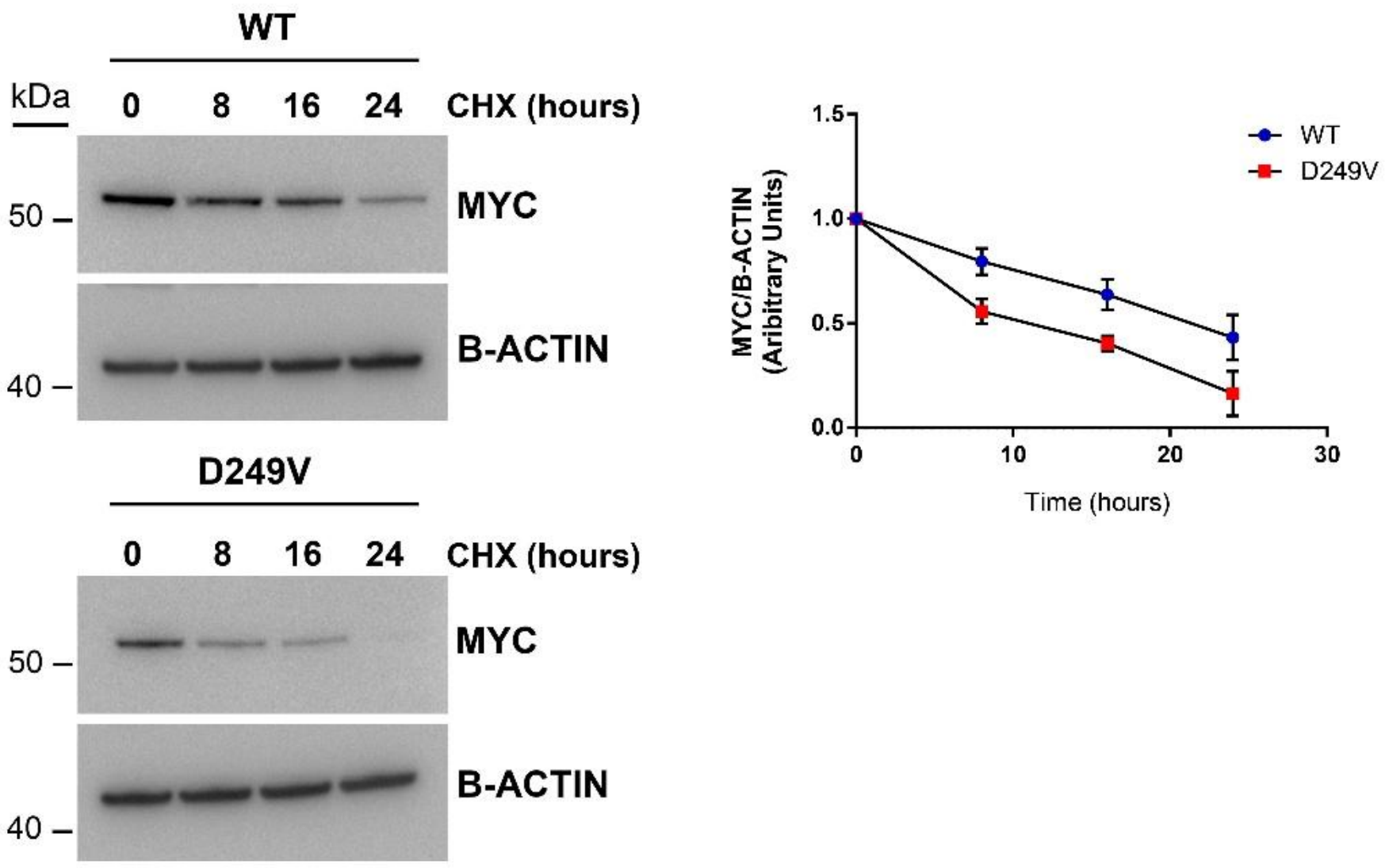

Supplementary Figure 6. The p.Asp249Val mutation affects TUBB short-term protein stability. HEK-293 cells transiently transfected with the MYC-tagged TUBB (WT and mutant) were treated with CHX, 24 hours after transfection and the turnover rates of TUBB were determined by western blotting at the indicated time points. On the right, a graphical representation of TUBB protein stability at different timepoints. Protein levels were normalized to β-actin and the relative protein intensities were compared to the 0 hour of WT or mutated TUBB. The graph reports the mean ± SEM. N=4. The difference between WT and mutant TUBB was not statistically significant (Mann Whitney test).

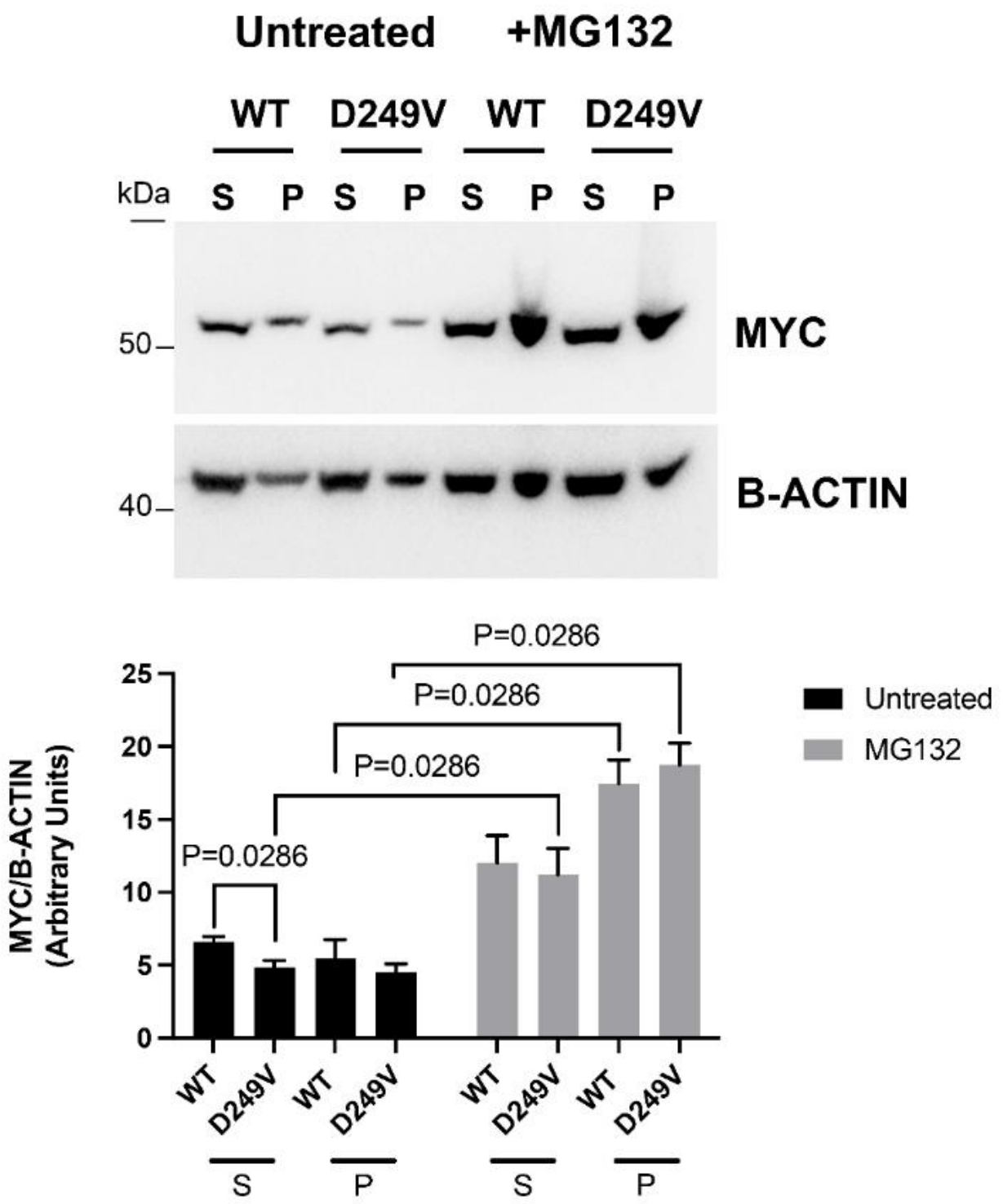

Supplementary Figure 7. Proteasome inhibition increases p.Asp249Val TUBB protein levels without altering its soluble and insoluble distribution. Immunoblot analysis of HEK-293 cells overexpressing WT or mutant MYC-tagged TUBB and treated for 8 hours with the proteasome inhibitor MG132 or DMSO (untreated) showed a significant increase in mutant TUBB levels in both the detergent-soluble (S) and detergent-insoluble (P) protein fractions, with no significant differences as compared to WT. β-actin was used as a loading control. The graph reports the mean ± SEM. N=4, Mann Whitney test.

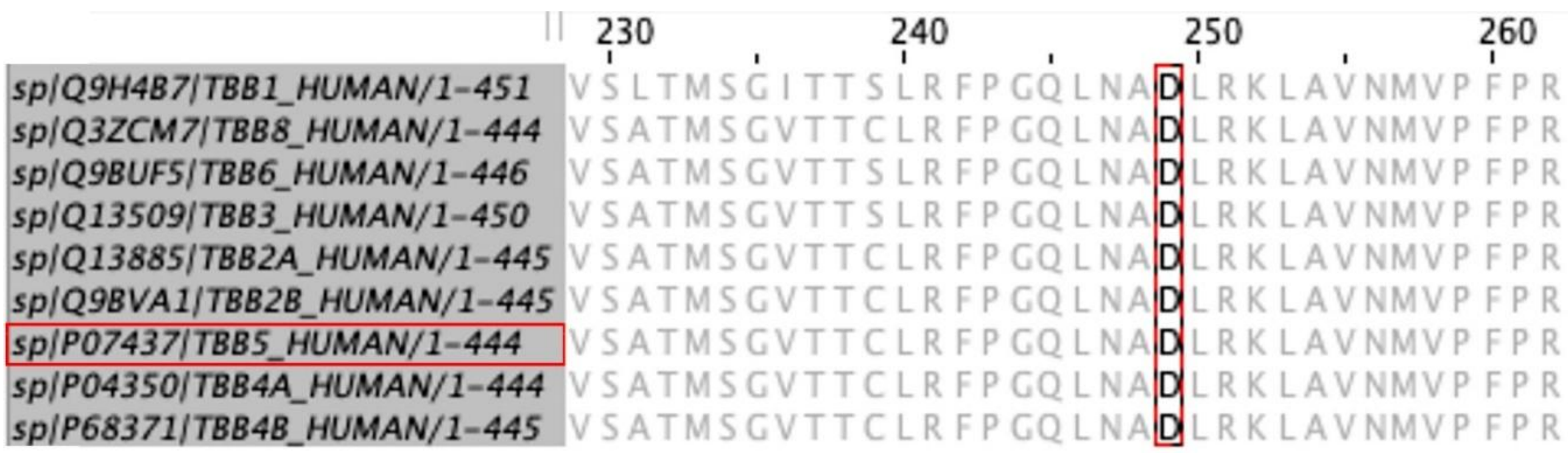

Supplementary Figure 8. *In silico* alignment of the amino acid variant p.Asp249Val. Multiple sequence alignment of β-tubulin protein isoforms around the site affected by the substitution p.Asp249Val associated with the pathological condition (red dashed box). Conserved residues, shown in grey, highlight strong conservation among the human paralogs in the region immediately adjacent to the residue of interest. In the alignment, *TBB5* represents an alternative gene symbol for *TUBB.* The alignment was performed using the Clustal Omega platform and visualized with Jalview.

Supplementary Table 1. List of Primers used in this study, related to Figure 3B.

Gene	Primer probe	Sequence
TUBB	Forward	5'-GGATCTGGGTGGAGGCGGTA- 3'
TUBB	Reverse	5'-GAACAGAGTCCATGGTCCCAG-3'
PGK1	Forward	5'-ATGGATGAGGTGGTGAAAGC-3'
PGK1	Reverse	5'-CAGTGCTCACATGGCTGACT-3'
HPRT	Forward	5'-TGACACTGGCAAAACAATGCA-3'
HPRT	Reverse	5'-GGTCCTTTTCACCAGCAAGCT-3'

PGK1 and *HPRT* were selected as reference genes based on [2].

Supplementary Table 2. List of Primers used in this study, related to Figure S3.

Gene	Primer probe	Sequence
TUBB	Forward	5'-ATACCTTGAGGCGAGCAAAA-3'
TUBB	Reverse	5'- ATCACCTCCCAGAACTTGGC-3'
PGK1	Forward	5'-ATGGATGAGGTGGTGAAAGC-3'
PGK1	Reverse	5'-CAGTGCTCACATGGCTGACT-3'
HPRT	Forward	5'-TGACACTGGCAAAACAATGCA-3'
HPRT	Reverse	5'-GGTCCTTTTCACCAGCAAGCT-3'

PGK1 and *HPRT* were selected as reference genes based on [2].

Supplementary Table 3. Main haematological parameters of the patient over time.

**Haematological Parameter**	**7 months**	**18 months**	**2 years, 6 months**	**4 years, 6 months**	**5 years, 6 months**
Hb (g/dL)	7.2	10.3	12.1	10.0	10.2
MCV (fL)	87.7	92	83.3	82.6	85
PLT (x10^3/mm^3)	95	43	52	15	23
MPV (fL)	8.9	7.7	9.5	7.2	8.2
WBC (x10^3/mm^3)	3.82	5.5	7.4	5.13	4.85
N (x10^3/mm^3)	0.22	1.36	2.15	2.53	2.32
anti-GPIIb/IIIa, anti-GPIa/IIa, anti-GPIb/IX	NA	Negative	Positive	NA	NA

Hb, haemoglobin; MCV, mean corpuscular volume; PLT, platelet count; MPV, mean platelet volume; WBC, white blood cell; N, neutrophil; NA, not available.

Supplementary Table 4. Gene list for inherited anaemia and bone marrow failure included in the NGS panel

ABCB7, ACD, ACTB, ADA2, ADAMTS13, AK1, AK2, ALAS2, AMN, ANK1, ANKRD26, AP3B1, AP3D1, ATM, ATR, ATRX, BLM, BLOC1S6, BRCA1, BRCA2, BRIP1, CASP10, CBL, CD40, CD40LG, CDAN1, CDIN1, CEBPA, CEBPE, CHEK2, CIITA, CLCN7, CLPB, CSF2RA, CSF3R, CTC1, CTLA4, CTSC, CUBN, CXCR2, CXCR4, DDX41, DHFR, DKC1, DNAJC21, DNASE2, DNMT3A, DPP9, EFL1, EIF2AK3, ELANE, EPB42,

EPCAM, EPO, ERCC4, ERCC6L2, ETV6, FANCA, FANCB, FANCC, FANCD2, FANCE, FANCF, FANCG, FANCI, FANCL, FANCM, FAS, FASLG, G6PC1, G6PC3, G6PD, GATA1, GATA2, GCLC, GFI1, GLRX5, GP1BA, GPI, GSS, HAX1, HFE, HLTF, HOXA11, HTRA2, HYOU1, IFNGR2, IKZF1, ITGA2B, JAGN1, JAK2, KDM1A, KIF23, KLF1, KRAS, LAMTOR2, LIG4, LPIN2, LYST, MAD2L2, MBD4, MECOM, MPIG6B, MPL, MRTFA, MTR, MYH9, MYSM1, NAF1, NBN, NF1, NHEJ1, NHP2, NOP10, NPM1, NRAS, NT5C3A, PALB2, PARN, PAX5, PC, PDHA1, PDHX, PGM3, PIEZO1, PKLR, POT1, PRF1, PTPN11, PUS1, RAB27A, RAC2, RAD23B, RAD51, RAD51C, RAD51D, RBM8A, RECQL4, REN, RFWD3, RFX5, RFXANK, RFXAP, RHAG, RMRP, RNF168, RPA1, RPL11, RPL15, RPL18, RPL26, RPL27, RPL31, RPL35, RPL35A, RPL5, RPL9, RPS10, RPS15, RPS17, RPS19, RPS24, RPS26, RPS27, RPS27A, RPS28, RPS29, RPS7, RTEL1, RUNX1, SAMD9, SAMD9L, SBDS, SBF2, SEC23B, SETBP1, SH2D1A, SLC11A2, SLC19A2, SLC25A38, SLC37A4, SLC4A1, SLX4, SMARCD2, SPTA1, SPTB, SRP54, SRP72, STAT3, STIM1, STK4, STX11, STXBP2, TAFAZZIN, TBXAS1, TCIRG1, TCN2, TERC, TERT, TET2, TF, THBD, TIMM50, TINF2, TMPRSS6, TP53, TPI1, TSR2, TUBA8, TUBB1, TYMS, UBE2T, UNC13D, USB1, VPS13B, VPS45, WAS, WDR1, WIPF1, WRAP53, XIAP, XRCC2, YARS2, ZCCHC8
