# Supplementary material for: Novel heterozygous truncating variant in TUBB associated with thrombocytopaenia and neurological abnormalities
Source: Orphanet J Rare Dis. 2026 Aug 13;21:415. doi: 10.1186/s13023-026-04546-5 (PMC13757095; doi:10.1186/s13023-026-04546-5)
Supplement: Supplementary file 2 [file 13023_2026_4546_MOESM2_ESM.docx]

**Uncropped files of Western Blot images**

**Uncropped files for Figure 3A**

**Uncropped files for Figure 3C**

**Uncropped files for Figure 4A**

**Uncropped files for Figure 5**

**Uncropped files for Figure 6A**

**Uncropped files for Figure 6B**

**Uncropped files for Supplementary Figure 2A**

**Uncropped files for Supplementary Figure 2B**

**Uncropped files for Supplementary Figure 3A**

**Uncropped files for Supplementary Figure 3B**

After detection of MYC, the membrane was probed with the anti-β-actin antibody and subsequently with the anti-GAPDH antibodiy. β-actin (45 kDa) signal remained visible after GAPDH (40 kDa) exposure.

**Uncropped files for Supplementary Figure 6**

**Uncropped files for Supplementary Figure 7**
